# Delayed Impact of Ionizing Radiation Depends on Sex: Integrative Metagenomics and Metabolomics Analysis of Rodent Colon Content

**DOI:** 10.3390/ijms26094227

**Published:** 2025-04-29

**Authors:** Nabarun Chakraborty, Gregory Holmes-Hampton, Matthew Rusling, Vidya P. Kumar, Allison Hoke, Alexander B. Lawrence, Aarti Gautam, Sanchita P. Ghosh, Rasha Hammamieh

**Affiliations:** 1 Medical Readiness Systems Biology, Center for Military Psychiatry and Neuroscience, Walter Reed Army Institute of Research, Silver Spring, MD 20910, USA; nabarun.chakraborty2.civ@health.mil (N.C.); matthew.r.rusling.mil@health.mil (M.R.); allison.v.hoke.civ@health.mil (A.H.); alexander.b.lawrence.ctr@health.mil (A.B.L.); aarti.gautam.civ@health.mil (A.G.); 2 Armed Forces Radiobiology Research Institute, Uniformed Services University of the Health Sciences (USUHS), Bethesda, MD 20889-5603, USA; gregory.holmes-hampton@usuhs.edu (G.H.-H.); vidya.kumar.ctr@usuhs.edu (V.P.K.); 3 Vysnova, Inc., Center for Military Psychiatry and Neuroscience, Walter Reed Army Institute of Research, Silver Spring, MD 20910, USA

**Keywords:** ionizing radiation, total body irradiation, sex-bias, metagenomics, metabolomics, descending colon content, chronic effect, time-course study

## Abstract

There is an escalating need to comprehend the long-term impacts of nuclear radiation exposure since the permeation of ionizing radiation has been frequent in our current societal framework. A system evaluation of the microbes that reside inside a host’s colon could meet this knowledge gap since the microbes play major roles in a host’s response to stress. Indeed, our past study suggested that these microbes might break their symbiotic association with moribund hosts to form a pro-survival condition exclusive to themselves. In this study, we undertook metagenomics and metabolomics assays regarding the descending colon content (DCC) of adult mice. DCCs were collected 1 month and 6 months after 7 Gy or 7.5 Gy total body irradiation (TBI). The assessment of the metagenomic diversity profile in DCC found a significant sex bias caused by TBI. Six months after 7.5 Gy TBI, decreased *Bacteroidetes* were replaced by increased *Firmicutes* in males, and these alterations were reflected in the functional analysis. For instance, a larger number of networks linked to small chain fatty acid (SCFA) synthesis and metabolism were inhibited in males than in females. Additionally, bioenergy networks showed regression dynamics in females at 6 months post-TBI. Increased accumulation of glucose and pyruvate, which are typical precursors of beneficial SCFAs coupled with the activated networks linked to the production of reactive oxygen species, suggest a cross-sex energy-deprived state. Overall, there was a major chronic adverse implication in male mice that supported the previous literature in suggesting females are more radioresistant than males. The sex-biased chronic effects of TBI should be taken into consideration in designing the pertinent therapeutics.

## 1. Introduction

A long history of radiation exposures caused by accidental, medical, environmental, or terrorist attacks has found a highly radiation-sensitive target, namely the gastrointestinal (GI) tract [1]. Radiation-induced damage to the GI tract emerged as a major chronic and acute subsyndrome of radiation injury that often results in fatality due to multisystem organ failure, sepsis, and complications due to bleeding. The highly sensitive nature of the GI tract is attributed to the fact that its epithelial barrier is made of rapidly dividing cells that could be easily damaged by irradiation, which can interrupt its cellular progression [1]. The compromised integrity of the GI tract usually leads to inflammation, the leaking of the colon contents into the bloodstream, and changing the ecosystem of the colon’s microbial community. Studying radiotherapy outcomes revealed that intestinal permeability is often restored within a week by the regenerative actions of surviving stem cells. However, dysbiosis in the intestinal microbial ecosystem has far-reaching consequences that have been associated with obesity [2] and multiple disease pathologies, including cancer [3,4], cardiovascular disorder [5], immune dysfunction [6], and several psychological illnesses [7,8]. There are multiple ongoing efforts that aim to restore the pre-stress healthy compositions of the gut microbiome [9,10,11,12] with the hope that the reverse engineering of the ecosystem would revitalize the host. The process of reverse engineering the microbial composition needs to take into account an array of cofactors, such as lifestyle alterations [13], age [14], and sex [15], since these cofactors can alter microbial composition [16,17,18].

The microbial community in and on the host body differs between males and females, where females host a larger abundance of microbial community than males; the ratio between the bacterial cells to host cells varies from 1.3 in the adult male to 2.2 in the adult female [19], and bacteria encompass the largest share of total abundance of resident microbes. This high microbial cellular load in adult females is attributed to a unique and complex ecosystem of microbes that is colonized in the female genital tract [20] and vagina [21,22]. In addition to the metagenomics reports, the multi-omics studies using animal models have indicated that ionizing radiation causes differential acute and chronic impacts on male and female cohorts [23,24]. This information potentially underscored the need to formulate a personalized medication for radiotherapy [25].

Age is another important cofactor that can control the microbial ecosystem [14]. For instance, microbial shifts that happen during pregnancy or childhood have been linked to many later health complications, such as obesity [26], diabetes [27], food allergies [28], and neurological disorders [29]. Among radiotherapeutic patients, gastrointestinal complications remain a long-term side effect and a major adverse effect on their quality of life [30].

Interestingly, a distinct set of microbes and microbial metabolites have been linked to those mice that survived irradiation, and a similar outcome was suggested within a small human cohort [31]. Hence, the evidence-supported hypothesis of the current study is that the shifts in the gut microbiome due to irradiation would differ between sexes, which might play a role in determining the chances of surviving lethal radiation.

Our past study suggested a near breakdown of the symbiotic activities between the host and the bacteria colonized in the rodent’s descending colon at 9 days after exposure to lethal radiation [32]. We postulated that at times of severe stress, when the resources of bioenergy become limited, but its demand escalates, the symbiotic relationship between the host and microbes becomes interrupted. At this stage, the microbes possibly enter their exclusive pro-survival mode, undermining the host’s health. A tangential support for this hypothesis was drawn from independent studies that reported higher longevity of the germ-free mice than that of the wild-type mice [33,34] since the germ-free mice were not obligated to share their biological resources with the microbes. Taking this observation into account, the present study was designed to understand the long-term impacts of radiation on the bacterial community colonized in descending colon contents (DCCs). In addition to screening the bacterial composition in DCC, we probed the metabolites extracted from DCC. Metabolites and biochemicals are the primary messengers that are shared within the communication grid, encompassing the ecosystem built upon the host and microbes [35,36,37,38]. For instance, the metabolites and biochemicals are involved in the majority of biological events that include genomic, epigenomic, and proteomic activities [35,36]; further, the metabolites are the typical intermediates of bionetworks and derivatives of the biological functions that take place in host cells and microorganisms alike [39]. There are around 200,000 metabolites in humans that are linked to nearly 1900 metabolic enzymes encoded in the genome [40]; on the other hand, there are merely 16,000 metabolites that are produced by microbes, of which about 11% are produced by microbes only, and rest of them are synthesized by both human and microbes [41]. Even though the host and microbes share a similar size of gene loads, the host produces more than 10 times the number of different metabolites, potentially due to the more complex biological functions that are undertaken by the host in comparison to the microbe. Two major classes of metabolites produced by microbiomes are secondary bile acid (SBA) and short chain fatty acids (SCFAs). In the present work, we have curated the microbiome-specific metabolites and pertinent biofunctions. By integrating this knowledge with sex-specific metabolites, we aimed to understand the sex-specific biomechanisms involved in responding to the long-term effects of radiation.

## 2. Results

**Figure 1:**
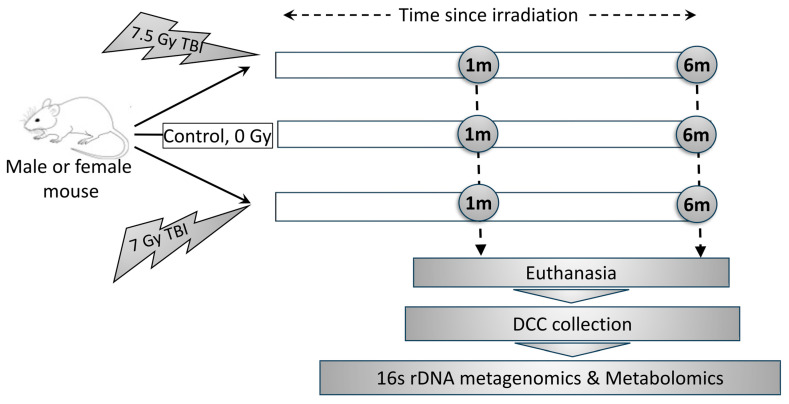
Outline of the research strategy. Mice were exposed to 7 Gy and 7.5 Gy total body irradiation (TBI), and their descending colon contents (DCCs) were collected at 1 month (1 m) and 6 months (6 m) post-TBI. In parallel, non-irradiated control’s DCCs were collected with age-matched 1 m and 6 m time periods. All DCC collection was conducted post-euthanasia.

C57BL/6 male and female mice were exposed to Co-60 gamma irradiation at two doses, namely 7 Gy and 7.5 Gy (Figure 1). Descending colon contents were collected 1 month and 6 months post-irradiation, sorted in two aliquots, and processed for 16S rRNA metagenomics and global metabolomics assays, respectively. Our previous communication [42] reported the body weight and complete blood count (CBC) of the mice that were exposed to a much wider dosimetry and were subsequently investigated for a long time range. From that data pool, we curated the parameters of current interest (e.g., 7 Gy and 7.5 Gy and the time points 1 m and 6 m post-irradiation) and processed them using the 3-way ANOVA model (Table S1). All blood cell types were found to be significantly depleted in the irradiated mice, and a majority of the cell types, including neutrophils, monocytes, red blood cells, and platelets, were differentially abundant (*p* < 0.05) between males and females (Table S1). The cumulative factor, namely Sex × RD, explained the variability in neutrophils and lymphocytes.

### 2.1. Sex-Biased Bacterial Diversity Profile

**Figure 2:**
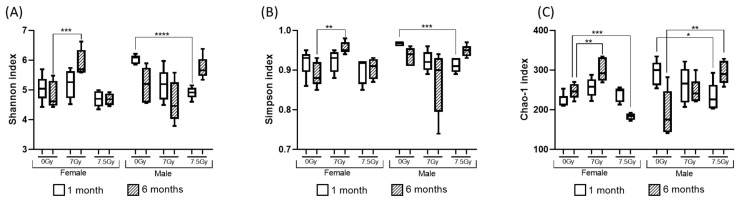

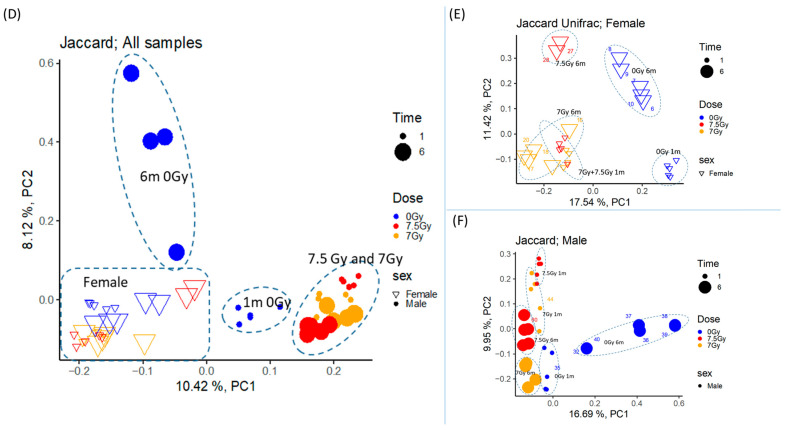
Diversity profile of the bacterial community in DCC. Figure (**A**–**C**): Alpha-diversity profile in box and whisker plots. Pertinent legends are at the bottom of each plot. Three-way ANOVA was computed to understand the impacts of sex, radiation dose (RD), and time since irradiation (TSI) on the alpha diversity profile. In these figures, changes in individual levels are captured by Welch’s *t*-test; **** *p* < 0.0001, *** *p* < 0.001; ** *p* < 0.01; * *p* < 0.05. (**A**) Shannon index; (**B**) Simpson index; (**C**) Chao-1 index. Figure (**D**–**F**): Beta-diversity profile in Principal Coordinate Analysis (PCoA) plot. Pertinent legends are on the right side of each plot. (**D**) All data points were plotted using a Jaccard index; subsequently, the male and female cohorts were plotted in (**E**) and (**F**), respectively.

The alpha diversity profiles of DCC microbes are presented in Figure 2A–C. Three-way ANOVA (Table 1) identified sex as the primary driving factor that caused alterations in alpha diversity. In addition, Sex × RD, Sex × TSI, and Sex × RD × TSI appeared as significant factors explaining alpha diversity. RD emerged as the leading cofactor that explained the significant changes in the Chao-1 index only. Further, alpha diversity was measured separately for male and female cohorts. For a male population, RD turned out to be the major cofactor explaining the shift in alpha diversity. Likewise, for the female population, RD turned out to be a major cofactor explaining the changes in Shannon and Simpson alpha diversity but not the Chao-1 index.

The box and whisker plots in Figure 2A–C depict sex-specific longitudinal trends in alpha diversity. In females, at 6 months post-TBI, the alpha diversity was significantly increased in the 7 Gy TBI cohort, but it was reduced in the 7.5 Gy TBI cohort. In the male cohort, a significant reduction in alpha diversity was noted 1 m post-radiation for both doses of radiation.

A PCoA plot using the Jaccard algorithm (Figure 2D) explains nearly 18.5% of beta diversity (PC1: 10.42% and PC2: 8.12% of the total variance). The samples were primarily clustered by sex. A 3-way PERMANOVA analysis (Table 1) using the Jaccard algorithm found sex and RD to be the leading cofactors in explaining beta diversity; *p* < 0.001 in both cofactors operating exclusively and cumulatively.

To understand the effects of sex, we re-plotted the male and female samples separately. Figure 2E displays the PCoA plot associated with females, where PC1 and PC2 explained 11.4% and 17.5% of the total variance, respectively. The samples were clustered in five groups; 7 Gy-1 m and 7.5 Gy-1 m are clustered together, and the rest of the samples, namely 0 Gy-1 m, 0 Gy-6 m, 7 Gy-6 m, and 7.5 Gy-6 m, are clustered separately. Radiation dose and its cumulative effects with time (RD × TSI) emerged as the primary factors explaining the beta diversity in females. This trend was nearly mirrored in the PCoA plot of the beta diversity in males (Figure 2F). Here, PC1 and PC2 explained 16.7% and 9.9% of the total variance, respectively, and the samples were clustered in six groups: for males, the 7 Gy-1 m and 7.5 Gy-1 m are separately clustered. RD and TSI both emerged as the driving forces to explain the beta diversity in the male population. Figure S1A–C presented a PCoA plot generated by a weighted Unifrac algorithm, and its clustering pattern was similar to the Jaccard algorithm (Figure 2D–F).

### 2.2. Differentially Abundant Taxa Under Bacterial Kingdom

The abundance profiles of all bacterial taxonomic levels are presented in Figure S2 and listed in Table S2. At the phylum level (Figure S2A), *Firmicutes* and *Bacteroidetes* aggregated maximum abundances, namely 57.3% and 37.0% of total phylum-level abundance. *Verrucomicrobia* and *Proteobacteria* were the third and fourth most abundant phyla, aggregating, on average, around 3.7% and 0.5% of total abundance. The remaining 1.1% of the abundance profile was populated by a combination of 21 phyla and unclassified entities.

A multiple comparative analysis (Table 2) of these four phyla found a significant sex bias (*p* < 0.05) in the shifting of the abundances of *Firmicutes* and *Verrucomicrobia.* Furthermore, the cumulative association between dose and time (RD × TSI) emerged as the significant cofactor (*p* < 0.05) causing the shift in the abundances of *Firmicutes* and *Verrucomicrobia* in the male cohort only.

In males, the abundances of *Bacteroidetes* (Figure S3B) and *Verrucomicrobia* (Figure S3C) significantly decreased from 1 m to 6 m post 7.5 Gy TBI, as these phyla were replaced by increasing abundance of *Firmicutes* from 1 m to 6 m post 7.5 Gy TBI (Figure S3A). In contrast, the abundance profile of *Firmicutes* showed no changes in females, although the abundance of *Verrucomicrobia* in females increased over time independent of the radiation dose.

In both male and female cohorts, the abundance shifts in *Bacteroidetes* were caused by the cumulative factor of RD × TSI. In females, the abundance profile of *Bacteroidetes* increased by RD × TSI, but this decreased in the male cohort, and a significant reduction was reported from 1 m to 6 m post-7.5 Gy TBI.

**Figure 3:**
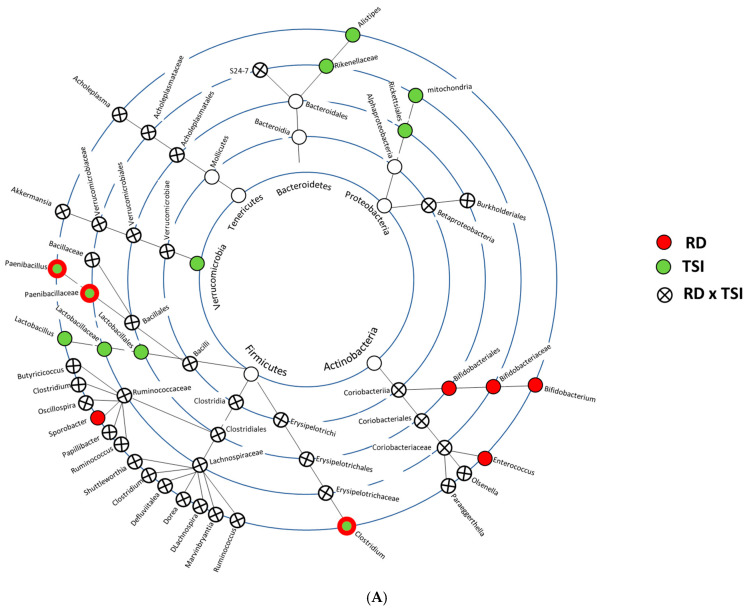

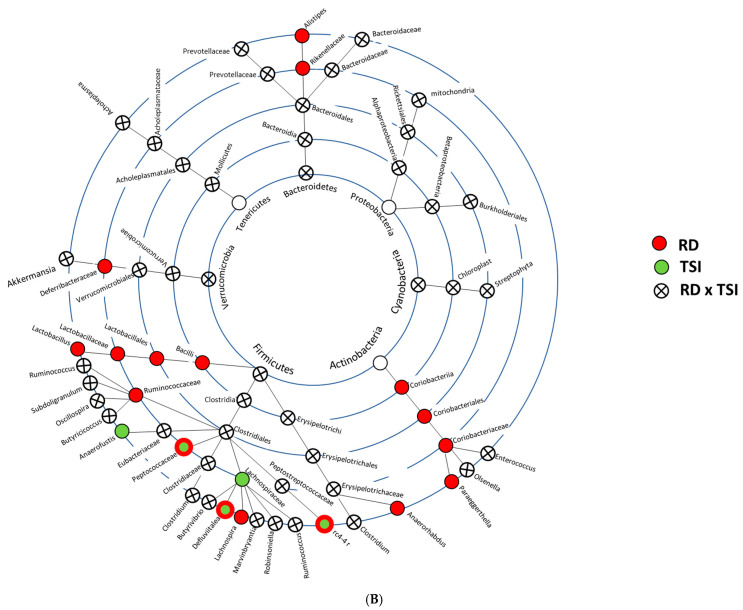
Cladograms showing the most perturbed bacterial taxa up until species level. The bacterial taxa, which were perturbed by RD and TSI, are presented here. The figure legends are on the left side of the plots. (**A**) Female- and (**B**) male-specific bacterial taxa that were significantly perturbed by RD and/or TSI.

Sex-specific LEfSe cladograms depicted those taxa that were differentially abundant due to radiation doses and/or time. Figure 3A,B depict female- and male-specific cladograms. Phylogenetic trees originating from *Firmicutes* and *Bacteroidetes* show shifts in abundance in both sexes, although, in males, the perturbations in *Bacteroidetes* were comparatively prominent and mostly driven by RD × TSI. Abundance shifts were reported in the phylogenetic tree that originated from a low-abundance phylum named *Actinobacteria*. In females, one of its branches stemming from the order *Coriobacteriales* showed a perturbation caused by RD × TSI, but the same branch in males was perturbed due to radiation only. Another low-abundant phylum, *Cyanobacteria*, showed perturbation only in males, and one of its branches was perturbed due to RD × TSI.

### 2.3. Differentially Perturbed Networks Linked to the Bacteria in DCC

Table S3A lists those biofunctions that were linked to the bacterial community colonized in the female descending colon. There were no common networks across all four experimental conditions, namely 1 month and 6 months post-7 Gy or 7.5 Gy TBI, respectively. Conversely, a majority of these networks (103) were perturbed at 6 months post-7 Gy TBI.

Likewise, there were 143 unique biofunctions that were differentially linked to the bacterial community (Table S3A) colonized in male descending colons, and 18 of these networks were common across all four experimental conditions, namely 1 month and 6 months post-7 Gy or 7.5 Gy TBI, respectively (Table S3B). A majority of these networks (107) were perturbed at 6 months post-7 Gy TBI.

**Figure 4:**
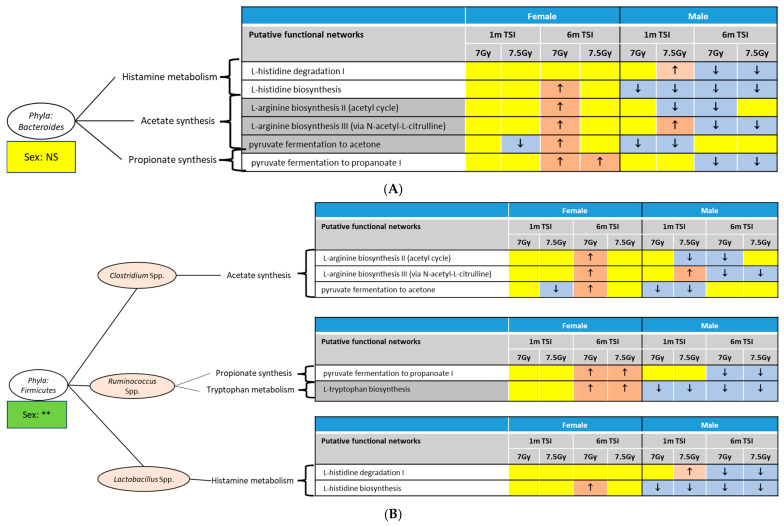
Integrative picture of bacterial taxa and associated networks. Here, the two most abundant phylum are presented. Corresponding networks had an LDA score of >|3.0|; the activated and inhibited networks are indicated by upward and downward arrows inside the red and blue boxes, respectively. The impact of sex on the phylum’s abundance is shown in the box at the bottom of the oval-shaped node containing the phylum’s name. (**A**) *Bacteroidetes* and associated bionetworks: The most enriched networks were the metabolism of amino acids, namely histamine, and the synthesis of SCFAs, namely acetate and propionate. Corresponding networks that were significantly enriched due to these assay parameters are presented. The abundance of *Bacteroidetes* was not differentially regulated between the sexes; hence, it is noted as NS or not significant. (**B**) *Firmicutes* and associated bionetworks: Under this phylum, *Clostridium* spp. and *Ruminococcus* spp. were significantly enriched due to RD × TSI in both sexes, and *Lactobacillus* spp. was significantly enriched due to TSI in females and RD in males. Corresponding networks that were significantly enriched are presented. The abundance of *Firmicutes* was differentially regulated between the sexes, ** *p* < 0.01.

All these abovementioned networks were classified under the following super families: metabolism of amino acids, carbohydrates, lipids, and xenobiotics, synthesis of SCFA and neurotransmitters, regulation of bioenergy, immune functions, and homeostasis of purine and pyrimidine compounds. Table 3 lists those microbiome-related networks that were differentially regulated by the cumulative factor (Sex × RD × TSI) in at least one of the four conditions, namely 7 Gy-1 m, 7 Gy-6 m, 7.5 Gy-1 m, and 7.5 Gy-6 m. Based on the currently available literature [39,43,44,45,46,47,48,49], Figure 4 integrates the functional networks with the major abundant phyla, *Bacteroidetes* (Figure 4A) and *Firmicutes* (Figure 4B). An overall low-abundant phylum, *Actinobacteria,* was differentially enriched between male and female mice; integrative functional analysis indicated that *Actinobacteria* is associated with lactate synthesis, which was largely inhibited in the male cohorts (Figure S4). Indeed, the study of the longitudinal regulation of biofunctions suggested a sex bias. For instance, in the male mice, the networks linked to acetate biosynthesis by *Bacteroidetes* and *Firmicutes* were mostly inhibited 6 m post-TBI.

### 2.4. Differentially Expressed Metabolites Derived from DCC as Markers of Host–Microbiome Association

The metabolite profile was analyzed to understand its responses to the cumulative impacts of three cofactors, namely sex, radiation dose, and time. The PCA of the entire metabolite landscape (Figure S5A) indicated that at 1 m post-7 Gy, male and female samples were clustered at two ends along the PC1, explaining 41.9% of the total variance. The overall trend of the metabolite profile shows a clear separation of the 1 month non-irradiated samples from the rest, which comprise the entirety of the groups of irradiated samples and 6 month non-irradiated samples.

**Figure 5:**
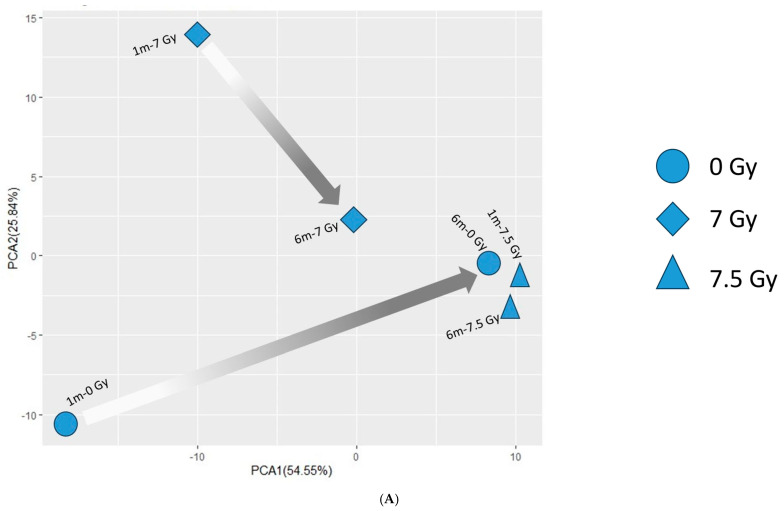

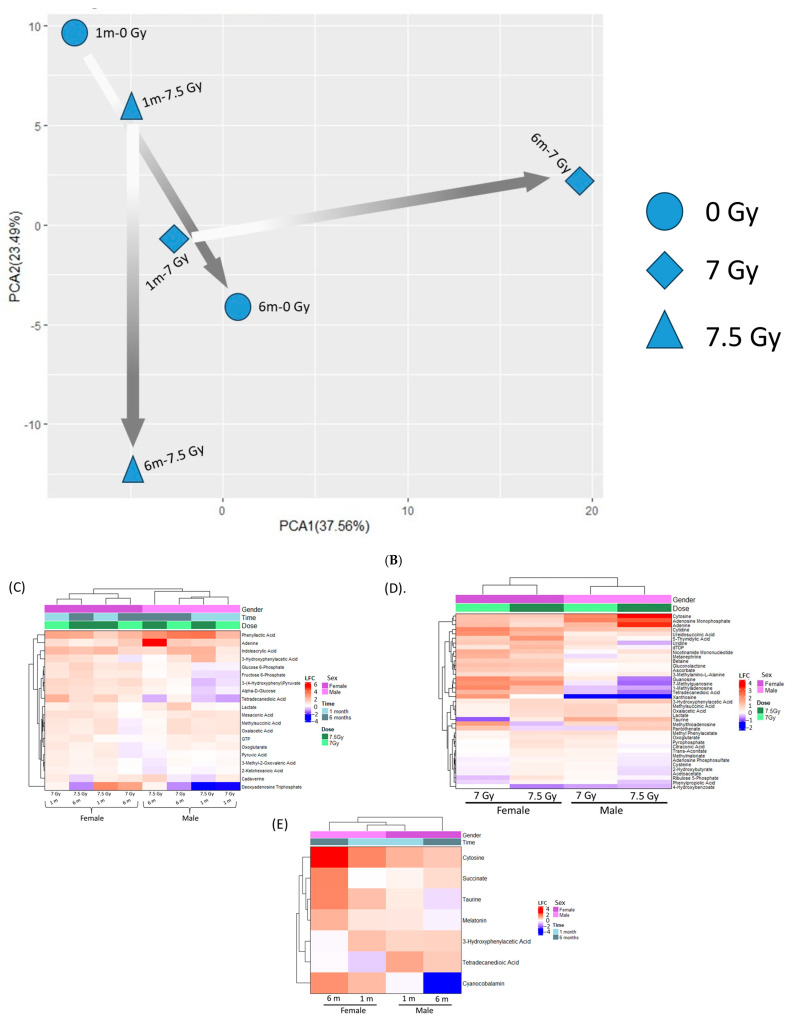
Fecal metabolite profile. Figure (**A**,**B**): Principal Component Analysis (PCA) plots show the distribution of the metabolite profile. The legends are on the right-hand side. The arrows trace the temporal shifts (1 m to 6 m) of those samples that were exposed to the same radiation doses. (**A**) Female- and (**B**) male-specific metabolite profile. Figure (**C**–**E**): Hierarchical clustering plots using the Euclidian algorithm show the differentially expressed metabolites (DEMs) identified by ANOVA. The legends are on the right-hand side. (**C**) Metabolites differentially expressed by Sex × RD × TSI. (**D**) Metabolites differentially expressed by Sex × RD. (**E**) Metabolites differentially expressed by Sex × TSI.

Male- and female-specific PCA plots (Figure 5A,B) reveal a similar pattern. The female-specific PCA of the metabolite regulation profile found that the baseline mice (0 Gy) and mice irradiated at 7 Gy were separated by a time difference (1 m → 6 m) across PC1 (54.5%); the temporal separation (1 m → 6 m) at 7.5 Gy TBI was minimum. In males, the temporal separations (1 m → 6 m) were observed in all radiation doses, namely 0 Gy or baseline, 7 Gy, and 7.5 Gy (Figure 4B).

Hierarchical clustering in Figure 5C–E depicts the impact of sex bias in conjugation with other cofactors, namely radiation dose and time. There were 20 metabolites that were differentially expressed by Sex × RD × TSI, and Figure 5C shows a clear dominance of sex in profiling these metabolites’ regulations. In addition, 39 and 7 metabolites were differentially expressed by Sex × Dose (Figure 5D) and Sex × TSI (Figure 5E), respectively, and in both cases, sex emerged as the dominating factor in profiling the metabolites. In addition, 38 metabolites were differentially expressed by RD × TSI (Figure S5B). Table S4 lists all these metabolites and a subset of the metabolites; those that showed consistent changes across the cofactors are shown in Table 4.

Table 5 indicates the biofunctions that were significantly enriched by the metabolites that were altered by cumulative cofactors, namely Sex × RD × TSI. In addition, Table S5A,B presents the networks enriched by those metabolites, which were altered by RD × TSI and Sex × RD. No networks met the significance threshold for Sex × TSI. Figure S6 shows a Venn diagram depicting the distribution of these networks across all the cofactors. There were no common networks among all three cumulative cofactors. Nevertheless, there were four networks, namely Production of reactive oxygen species, Cellular homeostasis, Concentration of lipid, and Insulin Secretion Signaling Pathway, common between RD × TSI and Sex × RD × TSI. A particular trend stood out in the regulation dynamics of Production of reactive oxygen species and Insulin Secretion Signaling Pathway (Table 5). Both networks were primarily activated 1 m post-TBI and inhibited 6 m post-TBI across both doses of irradiation. At the sex level, these networks were mostly activated in males, irrespective of radiation dose and time. In females, these networks were activated 1 m post-TBI and inhibited 6 m post-TBI across both doses of irradiation. In addition, there were four networks, namely Quantity of carbohydrate, Necrosis, Activation of cells, and Metabolism of nucleic acid component or derivative that were co-regulated by RD × TSI and Sex × RD. Likewise, the network linked to Quantity of amino acids was co-regulated by Sex × RD and Sex × RD × TSI.

## 3. Discussion

Our previous report studying a similar mouse model found a significant sex bias in survival from lethal radiation and came to the conclusion that female mice had an increased survival rate 6 months after total body irradiation [42]. Indeed, similar inferences have been suggested in the past, namely that female mice are potentially more resistant to irradiation [62,63,64], although the biological reason behind this potential sex bias is yet unclear. Meeting this knowledge gap, we focused on one aspect that differentiates males and females, namely the size and diversity of the microbial community that particularly colonizes inside the gut lumen [19,20,21].

An increasing number of studies have linked an array of host biofunctions to the microbial community that colonizes in and on the host [39,44]. For instance, the microbial roles in forging the host’s bioenergy [65] and other bio-expensive functions [44,66,67] have been documented. Our study showed that in moribund irradiated mice, the symbiotic relationship between the host and gut bacterial community broke down, as the gut bacterial community potentially forms a pro-survival environment exclusively for themselves [32]. Taken together, the current objective is to determine the long-term dose-dependent impact of irradiation on gastrointestinal (GI) health. Since gut microbial composition significantly differs between sexes, it was justified to conduct this research across the sexes and comprehend the metagenomic underpinnings to explain the female rodent’s higher radioresistant capability than that of its male counterpart.

The bacterial diversity profile of DCC showed different longitudinal shifts between the sexes. Alpha diversity, which measures the evenness and richness of microbial profile within a community [68], showed a sex-specific longitudinal shift for both radiation doses. Overall alpha diversity was reduced from the baseline in female mice that were exposed to 7.5 Gy; contrastingly, the alpha diversity in male mice increased at 6 m post 7.5 Gy TBI. A rather prominent sex bias was observed in the beta diversity. When measuring the trans-community occurrences, the Jaccard index showed clear distinctions between the irradiated male and female cohorts [38,68]. The Jaccard index-based diversity profile was mirrored by that measured by a weighted Unifrac that takes into account the abundances of different taxa [38].

As indicated by the beta diversity, the taxa-level abundance profile varied between the sexes in the context of time and dose. In the male cohort that was exposed to 7.5 Gy, the decreased abundances of *Bacteroidetes* and *Verrucomicrobia* were gradually replaced by the increased abundance of *Firmicutes*. There were not many phyla-level perturbations in female mice; nevertheless, the LefSe cladogram displayed a sex-based variability in the high-level taxa. Briefly, the *Firmicutes* branch showed major perturbation in both sexes, and the perturbations of sub-branches like *Firmicutes–Clostridia–Clostridiales–Clostridiaceae–Clostridium* were mostly conserved between the sexes. On the other hand, a phylum named *Cyanobacteria* was only perturbed in the irradiated male cohorts.

These microbes undertake very specific biofunctions; hence, any sex bias in the microbial abundance profile directly impacts the biofunctions. For instance, *Bacteroidetes* are primarily linked to the synthesis of SCFAs, like butyrate, propionate, and acetate [47]. Reduced abundance of *Bacteroidetes* in irradiated males was manifested by inhibited networks linked to SCFA generation. The *Clostridium* species in the *Firmicutes* branch [39,69] is also responsible for acetate generation, and its reduced abundance in males was further manifested by the inhibited networks of acetate synthesis. Likewise, inhibited propionate metabolism networks were linked to the male DCC *Ruminococcus* spp. under the *Firmicutes* phylum [39,70].

There was an overall inhibition of SCFA biosynthesis in the irradiated male mice but not in the female mice. In conjugation, the metabolomics assay found upregulated pyruvate abundance in the irradiated male mice. As the precursor of all major SCFAs, pyruvate produces acetate and butyrate via an acetyl co-A intermediate and generates propionate via the succinate pathway [71]. Lactate and its derivatives, like propionate, are additional downstream products of pyruvate metabolism. Upregulated pyruvate in DCC suggested a dysbiosis, with inhibited synthesis of some of the major SCFAs. This observation is particularly important in the context that the high concentrations of three SCFAs, namely acetate, butyrate, and propionate, in the gut lumen were observed in mice that survived lethal irradiation [31]. The same study identified tryptophan in the gut lumen as an additional survival marker [31]. Our study found a rather clear, distinctive profile of the pertinent networks in male vs. female mice. In the irradiated male cohorts, the networks linked to propionate synthesis and tryptophan metabolism were mostly inhibited; however, they were activated or remained unchanged in females.

Inhibited SCFA synthesis often co-occurred with the accumulation of lactate in the gut milieu as a sign of dysbiosis [45], and we observed a similar result in the irradiated males only. Lactate is the primary precursor of one of the major SCFAs, namely propionate. There was a diverging trend in the regulation profiles of the networks linked to propionate synthesis (inhibited in males) and lactate synthesis (activated in males). A somewhat contrasting picture emerged in the female groups, where activated networks linked to propionate synthesis were aligned to inhibited networks linked to lactate synthesis. In a healthy gastrointestinal lumen, lactate and pyruvate are typically used for cross-feeding for SCFA production [72]; thus, the concentrations of these metabolites are typically low [73]. Increased accumulation of pyruvate and lactate in the gut lumen could be triggered by the systematic replacement of *Firmicutes* and *Bacteroidetes* by lactate-producing microbes [45,72]. Positively, our study reported an increased aggregation of the lactate-producing phylum *Actinobacteria* in irradiated males 1 month post-TBI, although it sharply decreased at 6 months post-TBI.

Overall, the metabolite profile of the DCC in the current study showed a time-dependent and less sex-biased distribution. Hypothetically, the gut metabolite landscape captures both the host and microbial response to irradiation [38]; therefore, it could be postulated that the host–microbe cumulative response to radiation was primarily controlled by the delayed time point, and it thereby took a diverging pattern from microbial response dynamics, which was primarily controlled by sex. The differentially expressed metabolite profile suggested time-/sex-/dose-independent radiation biomarkers, such as phenyllactic acid, which is a microbial metabolite synthesized via the metabolism of phenylalanine; this metabolite has been previously identified as a marker of radiation-induced liver injury [50]. Indeed, the higher load of phenyllactic acid, glucose, and pyruvate across the sexes indicates metabolism disorder and bioenergy deprivation. All these chemicals are typical inputs or intermediates of metabolism chains and the components for cross-feeding across microbes to produce energy; hence, these molecules are low in abundance in healthy hosts.

Activated reactive oxygen species (ROS) producing networks in the DCC metabolite analysis further suggest an energy-deprived condition in the hosts [74]. In the females irradiated at 7 Gy TBI, the activated network linked to ROS production had a long-term impact; in contrast, in the irradiated male mice, the increased ROS production at 1 m post-TBI returned to baseline levels at 6 m post-TBI. Increased ROS concentration in the intestinal lumen causes long-term dysbiosis, which was suggested by the comprehensive inhibition of metabolism of SCFA synthesis in the irradiated male mice. Again, there was a sign of divergence in the female microbial functional profile, as the inhibited SCFA metabolism was found only at 1 month post-7.5 Gy TBI. In the rest of the samples, these networks were activated concurrently with bioenergy-producing networks; all these potentially indicate a regression dynamic exclusive to the irradiated female cohorts.

The current study is limited by the sample depth; to compensate, we employed a multi-omics assay and comprehensive integrative model. Further, we presented 16s rRNA metagenomics data that, despite recent advancements in pertinent analytical pipelines, have a few inherent limitations. For instance, the resulting data have limited power in terms of confidence to explain beyond the family level. Since the current study is mostly focused on the bacterial community, there was a limited scope in drawing inferences about cross-kingdom communication in response to stress. Nevertheless, the present study was able to suggest potential sex-specific mechanisms to explain the long-term impacts of lethal radiation exposure. We observed a significantly altered microbial ecosystem 1 month and 6 months post-TBI in a murine model, which was nearly equivalent to 3 years and 18 years of human lives, respectively [75]. Even after such a long recovery period, the host–microbiome association was apparently still damaged and rather diverged between male and female mice. Chronic inhibition of SCFA synthesis in the irradiated males but not in the irradiated female mice is a potential explanation for the comparatively high radioresistant characteristics of female mice. The present data could be instrumental in developing next-generation customized medication strategies as studies begin promoting the concept that radiotherapy would be more effective should the sex factor be taken into account [25].

## 4. Materials and Methods

### 4.1. Animals

Pathogen-free male and female C57BL/6 mice (11–14 weeks old) were purchased from Jackson Laboratories (Bar Harbor, ME, USA). Animals were housed as reported previously [42,76] in the Uniformed Services University of the Health Sciences (USUHS) Department of Laboratory Animal Resources (DLAR) facility and acclimated for a minimum of 5 days prior to use in experiments. All animals were identified by unique tail tattoos. Both room and cage humidity were maintained between 30–70%, and 10–15 air changes/hour occurred in the housing room. An automated lighting system was used, providing a 12 h light, 12 h dark cycle. Mice were provided Harlan Teklad Global Rodent Diet 8604 ad libitum from the feeder rack within the cage. The water provided was acidified pH~2.5 from an Edstrom water bottle filling station. All procedures related to animal manipulation were reviewed and approved by the USUHS Institutional Animal Care and Use Committee (IACUC) using the principles outlined in the National Research Council’s Guide for the Care and Use of Laboratory Animals and performed in accordance with relevant guidelines and regulations. Animal studies were conducted in compliance with ARRIVE (Animal Research: Reporting of In Vivo Experiments) guidelines. Handling of the animals was conducted in accordance with the USUHS IACUC policy. Animals were examined by the veterinary staff as well as laboratory staff, as warranted by clinical signs or changes in appearance [76].

### 4.2. Whole-Body Gamma Irradiation

The animals were transported in a climate-controlled van to the Armed Forces Radiobiology Research Institute (AFRRI) Co-60 gamma irradiation facility. After arrival, they were rested for a minimum of 60 min prior to irradiation in custom-made Lucite restrained boxes with 8 compartments. After radiation exposure, animals were returned to their cages and ultimately returned to the DLAR facility via the climate-controlled van. Non-anesthetized mice were irradiated bilaterally (simultaneously) at an estimated dose rate of 0.6 Gy/min. An alanine/Electron Spin Resonance (ESR) precise dosimetry system (American Society for Testing and Material Standard E 1607) was used as described earlier [76].

### 4.3. Ethics Statement and Veterinary Care Following Radiation

This study was conducted under an animal use protocol approved by the USUHS IACUC, Protocol Number: AFR-20-999, following the USDA Animal Welfare Act (21 CFR Part 9) and Public Health Service Policy, the Guide for the Care and Use of Laboratory animals, and the Office of Laboratory Animal Welfare, as applicable. The Testing Facility is accredited by the Association for the Assessment and Accreditation of Laboratory Animal Care (AAALAC) International.

Since animals were irradiated from non-lethal to sub-lethal doses, they were monitored three to four times daily following exposure [76]. The model used does not include supportive care in the form of analgesia. Animals that were found dead in the course of the study were documented and removed from the cage. Mice were considered moribund when they exhibited certain symptoms, including an inability to remain upright, were cold, unresponsive, or displayed decreased or labored respiration. Morbid animals were monitored very closely according to their health condition in accordance with pre-defined criteria described and approved in the IACUC protocol. Moribund mice were euthanized according to American Veterinary Medical Association (AVMA) guidelines.

### 4.4. Post-Euthanasia Sample Collection

Mice were exposed to 7 Gy and 7.5 Gy total body irradiation (TBI), and their descending colon contents (DCC) were collected at two time points, namely 1 month (1 m) and 6 months (6 m) post-TBI. In parallel, non-irradiated (0 Gy) control’s DCC were collected with age-matched 1 m and 6 m time periods. Each group included 5 mice. Post-euthanasia, the entire intestine was incised on ice; the DCCs were removed from the descending colon tissues, cryogenically homogenized, and subsequently stored at −80 °C (freezer) for long-term storage. On the day of sample extraction, the DCCs were aliquoted for 16S rRNA metagenomics gene sequencing and metabolomics assays, respectively.

### 4.5. 16S rRNA Sample Processing Using Descending Colon Contents

DCC samples were divided into two aliquots: one for the 16S rRNA metagenomics assay, and the other one was assigned to metabolomics assay. The PowerSoil DNA Isolation Kit (MoBio Laboratories, Inc., Carlsbad, CA, USA) was used to extract DNA from DCCs following our previously published method [32,77,78]. The extracted DNA from DCCs was used for the 16S rRNA gene sequencing study following the guidelines provided by the Illumina 16S Metagenomics Library Preparation manual (Illumina, Inc., San Diego, CA, USA). Briefly, a set of pre-designed primers extracted the hyper-variable V3 and V4 regions of the 16S rRNA amplicon [79]. The samples were barcoded using Nextera indexes, and target amplicons of nearly 460 bases long were subsequently generated. The libraries were pooled and sequenced on the Illumina MiSeq platform, using paired-end 300 bp reads and Illumina MiSeq v3 reagents. The end of each read was overlapped to generate high-quality, full-length reads of the V3 and V4 regions.

### 4.6. 16S rRNA Metagenomics and Functional Network Analysis

This analysis was conducted following our previously published protocols [32,78,80]. The raw demultiplexed FASTQ files were imported into R and processed using the DADA2 [81] package from Bioconductor [82], following the standard procedure on demultiplexed sequences. Briefly, the reads were inspected for quality, filtered, and truncated at 220 bp; the paired reads were merged, a sequence table of amplicon sequence variants (ASVs) was constructed, and chimeras were removed. The ASV table was imported into QIIME2 v.2019.7 [83].

Alpha diversity was measured using the Simpson [84], Chao1 [85], and Shannon [86] indices. Two-way ANOVA was computed in R Studio (R version 2022.12.0) to determine alpha group significance across the cofactors, namely radiation dose (RD) and time since irradiation (TSI); the significance value cutoff was *p* < 0.05. Beta diversity was calculated using the Bray-Curtis and Euclidean algorithm [87], and the Principle Coordinate Analysis (PCoA) was estimated using q2-diversity. The Adonis plugin in QIIME2 [88] computed a two-way permutational multivariate analysis of variance (PERMANOVA), where the two cofactors were RD and TSI, respectively, with the significance cutoff at *p* < 0.05. To note, all the subsequent multi-factorial comparison analyses used these two cofactors, namely RD (7.0 Gy vs. 7.5 Gy) and TSI (one month and six months post-TBI), and their cumulative model, e.g., RD × TSI.

The taxonomic classification of the ASVs was generated by a q2-feature-classifier [89] via the classify-sklearn plugin [90] using the GreneGenes reference database (13-8-99-515-806 classifier). Next, linear discriminant analysis effect size (LEfSe) curated the top-ranked taxonomic classifiers that discriminated 7 Gy from 7.5 Gy at three time points post-TBI using the *lefser* package (R package version 1.14.0), and subsequently, the cladogram plotted those taxa, which met cutoff LDA > |2| [91]. Further, Phylogenetic Investigation of Communities by Reconstruction of Unobserved States (PICRUSt2) was used to predict the MetaCyc pathway abundances using the sequencing-derived taxonomic ASVs to generate the functional profile—a list of networks that are potentially enriched by microbial metabolites; here, the non-irradiated CTR mice were used as the global baseline in LEfSe with cutoff LDA >|2| [92,93,94,95,96].

### 4.7. Global Metabolomics Assay of DCC

The second aliquot of DCC samples was used for untargeted metabolomics profiling following our standard protocol [97,98]. For the metabolomics sample preparation, 150 µL of DCC solution was mixed with internal standards containing 5 mL water, 5 mL methanol, 10 µL debrisoquine (1 mg/mL in ddH_2_O), and 50 µL of 4-nitrobenzoic acid (1 mg/mL in Methanol) (per 10 mL). Further, 150 µL of chilled (~20 °C) acetonitrile was mixed with the ice-cold metabolomics samples and incubated together at −20 °C for 20 min. Lastly, the samples were centrifuged at 15,493× *g* for 20 min at 4 °C, and the supernatant was transferred to an MS vial for LC-MS analysis. Two microliters of each prepared sample was injected onto a Waters Acquity BEH C18 1.7 μm, 2.1 × 50 mm column using an Acquity UPLC system coupled to a Xevo G2-S quadrupole-time-of-flight mass spectrometer with an electrospray ionization source (UPLC-ESI-QToF-MS) (Waters Corporation, Milford, MA, USA). The mobile phases consisted of 100% water (solvent A), 100% acetonitrile containing 0.1% formic acid (solvent B), and 100% isopropanol with 0.1% formic acid (solvent C). The solvent flow rate for the metabolomics acquisition was set to 0.4 mL/min, with the column set at 60 °C. The LC gradient was as follows: Initial—95% A, 5% B; 0.5 min—95% A, 5% B; 8.0 min—2% A, 98% B; 9.0 min—11.8% B, 88.2% C; 10.5 min—11.8% B, 88.2% C; 11.5 min—50% A, 50% B; 12.5 min—95% A, 5% B; 13.0 min—95% A, 5% B. The column eluent was introduced into the Xevo G2-S mass spectrometer by electrospray operating in either negative or positive electrospray ionization mode. Positive mode had a capillary voltage of 3.00 kV and a sampling cone voltage of 30 V. Negative mode had a capillary voltage of 2.00 kV and a sampling cone voltage of 30 V. The desolvation gas flow was set to 600 L/h, and the desolvation temperature was set to 500 °C. The cone gas flow was 25 L/h, and the source temperature was set to 100 °C. The data were acquired in the sensitivity MS mode with a scan time of 0.300 s and an interscan time of 0.014 s. Accurate mass was maintained by infusing Leucine Enkephalin (556.2771 [M + H]^+^/554.2615 [M − H]^−^) in 50% aqueous acetonitrile (2.0 ng/mL) at a rate of 10 µL/min via the Lockspray interface every 10 s. The data were acquired in centroid mode with a 50.0 to 1200.0 m/z mass range for TOF-MS scanning. An aliquot of each sample was pooled and used as a quality control (QC), which represented all metabolites present.

The spectral features acquired herein were first converted to the NetCDF unified data format using the Databridge tool in MassLynx (Waters Corporation, Milford, MA, USA). The XCMS R package (Scripps Institute, La Jolla, CA, USA) was used for peak detection, and the interpolated warping algorithm was utilized for retention time correction and parameters optimized using the Isotopologue Parameter Optimization (IPO) R package. [99] The mass-to-charge ratio and retention time features were normalized based on the internal standards (debrisoquine and 4-nitrobenzoic acid present in the extraction solution in positive and negative modes, respectively) as well as QC-RLSC (QC robust LOESS signal correction) normalization.

### 4.8. Functional Metabolomics Analysis

The spectral features were analyzed by repeated measures two-way ANOVA with three cofactors, namely sex (male and female), time (one month and six months post-TBI), and radiation dose (7 Gy and 7.5 Gy). The spectral features that scored above the threshold of *p* < 0.05 were curated as the significantly differential peaks for the following four experimental parameters, namely Sex × Time since irradiation (Sex × TSI), Sex × Radiation Dose (G × RD), Time since irradiation × Radiation Dose (TSI × RD) and Sex × Time since irradiation × Radiation Dose (G × TSI × RD). The differentially expressed spectral features were annotated using the CEU Mass Mediator 3.0 database (https://ceumass.eps.uspceu.es/, accessed on 4 April 2025), and the molecules were screened based on the following guidelines [97,98]: (a) ppm error < 1; (b) chemical formula comprised of the adducts: +H, −H, +Na, +K, +NH4, and −Cl; (c) annotation by Human Metabolome Database (HMD); and (d) chemical type of one of the following categories: (i) endogenous mammalian; (ii) drugs; (iii) toxicant; (iv) reagents. Thereby, we identified the differentially expressed metabolites (DEMs). Annotated metabolites were seeded into Ingenuity Pathway Analysis (IPA, QIAGEN, Germantown, MD, USA) to curate the networks with a cutoff z score > |1.5|.

## Figures and Tables

**Table 1:** Diversity profile analysis to understand the impacts of various cofactors, namely sex (male and female), dose (TBI doses, 7 Gy and 7.5 Gy), and time (time since irradiation, 1 month and 6 months post-TBI), on alpha and beta diversity. This table shows the levels of significantly altered diversity within and across the bacterial community in DCC. The diversity values are displayed in Figure 2. NS: non-significant; * *p* < 0.05; ** *p* < 0.01; *** *p* < 0.001; crossed box: non-applicable cofactor; RD; radiation dose; TSI: time since irradiation.

Diversity	Index	Cohort	Sex	RD	TSI	Sex × RD	RD × TSI	Sex × TSI	Sex × RD × TSI
Alpha	Shannon	All	**	NS	NS	***	**	NS	**
		Male	--	***	NS	--	*	--	--
		Female	--	**	NS	--	**	--	--
	Simpson	All	*	NS	NS	***	*	NS	*
		Male	--	*	NS	--	NS	--	--
		Female	--	*	NS	--	*	--	--
	Chao-1	All	**	*	NS	**	*	NS	***
		Male	--	***	NS	--	***	--	--
		Female	--	NS	NS	--	***	--	--
Beta/PERMANOVA		All	***	***	*	***	*	***	***
		Male	--	**	NS	--	**	--	--
		Female	--	***	***	--	***	--	--

**Table 2:** Impacts of different cofactors, namely sex (male and female), dose (TBI doses, 7 Gy and 7.5 Gy), and time (time since irradiation, 1 month and 6 months post-TBI) on the abundance profile of different phyla. This table shows the levels of significantly shifted bacterial phylum level abundance. The abundance profile of these bacterial taxa is reported in Figure S3. NS: non-significant; * *p* < 0.05; ** *p* < 0.01; *** *p* < 0.001; crossed box: non-applicable cofactor; RD; radiation dose; TSI: time since irradiation.

Phylum	Cohort	Sex	RD	TSI	Sex × RD	RD × TSI	Sex × TSI	Sex × RD × TSI
*Bacteroidetes*	All	NS	NS	NS	NS	NS	*	NS
	Male	**--**	NS	NS	**--**	*	**--**	**--**
	Female	**--**	NS	NS	**--**	**	**--**	**--**
*Firmicutes*	All	**	NS	NS	NS	NS	**	**
	Male	**--**	NS	*	**--**	*	**--**	**--**
	Female	**--**	NS	NS	**--**	NS	**--**	**--**
*Proteobacteria*	All	NS	NS	*	NS	NS	NS	NS
	Male	**--**	NS	NS	**--**	NS	**--**	**--**
	Female	**--**	NS	NS	**--**	NS	**--**	**--**
*Verrucomicrobia*	All	***	NS	NS	NS	NS	NS	***
	Male	**--**	NS	NS	**--**	*	**--**	**--**
	Female	**--**	NS	*	**--**	NS	**--**	**--**

**Table 3:** Superfamilies of the putative networks linked to bacteria in DCC. The networks with an LDA score of >|3|. Each cell includes the number of activated networks and the number of inhibited networks, separated by a hyphen (“/”). The cells are colored red, green, and white, representing the number of activated networks that were more, less, or equal to the number of inhibited networks, respectively.

	Female				Male			
	1 Month TSI		6 Month TSI		1 Month TSI		6 Month TSI	
Superfamilies of Networks	7 Gy	7.5 Gy	7 Gy	7.5 Gy	7 Gy	7.5 Gy	7 Gy	7.5 Gy
Carbohydrate metabolism	1/0	0/0	6/0	3/0	0/4	2/4	0/8	0/4
Lipid metabolism	0/0	0/3	2/1	0/0	1/0	3/3	0/5	0/5
Amino acid metabolism	5/0	0/8	38/4	10/0	1/17	9/27	1/35	4/22
SCFA biosynthesis	0/0	0/2	9/0	4/0	0/4	1/6	0/8	0/66
Purine and pyrimidine homeostasis	1/0	0/8	2/8	1/0	1/0	1/5	0/19	0/12
Bioenergy	0/0	0/0	11/3	4/0	5/0	12/1	4/9	1/7

**Table 4:** A subset of the differentially expressed metabolites that showed sex bias in response to TBI. The list is sorted into two groups. (i) The metabolites that were changed by the cumulative association among all three cofactors are listed under Sex × TSI × RD. (ii) The metabolites that were changed by the cumulative association between Sex and RD are listed under Sex × RD. There were not many features that changed due to Sex × TSI. ♀: Female; ♂: Male.

Sex × TSI × RD		
Metabolite	Regulation Status	Study Relevance
Phenyllactic Acid	♀ + ♂: upregulated.	A microbial metabolite previously identified as the marker of radiation-induced liver injury [50]. *Potential sex-/dose-/time-independent radiation marker.*
Adenine	♀ + ♂: upregulated.	Adenine contributes to host purine homeostasis and supports the growth of *Proteobacteria* and *Firmicutes* [51]. Adenine inhibits intestinal epithelial mucosal inflammation [52]. As a purine base, it is a precursor of nucleic acid in intestinal cells and markers of DNA damage. Additionally, it is produced by *E.coli*. *Potential sex-/dose-/time-independent radiation marker.*
Indoleacrylic Acid	♀ + ♂: upregulated.	Intestinal microorganisms catabolize tryptophan to indoles, which are converted into indoleacrylic acid, an anti-inflammatory agent that helps in bolstering the intestinal barrier [53]. *Potential sex-/dose-/time-independent radiation marker.*
Tetradecanedioic Acid	♀: upregulated. ♂: downregulated, except 6 m-7 Gy upregulated.	Related to glucose metabolism and radiation exposure [54].
Glucose	♀: upregulated. ♂**:** For all RD, 1 m downregulated; 6 m upregulated.	Radiation exposure is typically linked to the malabsorption of glucose [55].
Pyruvate	♀: upregulated. ♂: For all RD, 1 m downregulated; 6 m upregulated.	Typically, a low abundance of SCFA that are shared among different microbes for cross-feeding [56,57]; hence, its high abundance is a marker of dysbiosis.
Deoxyadenosine Triphosphate (dATP)	♀: In 7 Gy, regulation status shifted to upregulated with time. Contrastingly, in 7.5 Gy, regulation status shifted to downregulated with time. ♂: Mostly downregulated.	Reduced dATP is a marker of mitochondrial dysfunction.
**Sex** × **RD**		
Cytosine	♀ + ♂: upregulated.	Markers of DNA damage. *Potential sex-/dose-independent radiation marker.*
Adenosine Monophosphate (AMP)	♀ + ♂: upregulated.	An overloaded AMP molecule, along with a depleted ATP concentration, is a signature of an energy-deprived condition [58]. In the present condition, we found a reduced concentration of dATP. *Potential sex-/dose-independent radiation marker.*
1-Methyladenosine	♀: upregulated. ♂: downregulated.	Biomarker of tumors. *Potential sex-specific radiation marker.*
Xanthosine	♀: upregulated. ♂: downregulated.	Important marker of host purine homeostasis [51]. *Potential sex-specific radiation marker.*
Taurine	♀: switched from 7 Gy (downregulated) to 7.5 Gy (upregulated) ♂: upregulated.	A bile acid component that helps mitigate gut permeability [59], although a high load could cause gastrointestinal discomfort.
Methylthioadenosine (MTA)	♀: switched from 7 Gy (upregulated) to 7.5 Gy (downregulated) ♂: upregulated.	Participates in purine salvage pathway and suppresses tumorigenesis [60].
Guanosine	♀: upregulated. ♂: switched from 7 Gy (upregulated) to 7.5 Gy (downregulated).	Neuroprotective agent against ischemic damage [61].
Pantothenate	♀: switched from 7 Gy (upregulated) to 7.5 Gy (downregulated). ♂: switched from 7 Gy (downregulated) to 7.5 Gy (upregulated).	Agent to form coenzyme-A (CoA); hence, it is critical in the metabolism and synthesis of carbohydrates, proteins, and fats.

**Table 5:** DCC metabolite-enriched bionetworks that were differentially perturbed by the cumulative impacts of all three cofactors, namely sex, radiation dose, and time (Sex × RD × TSI). Networks that scored |z| > 1.5 in any of the featured condition are reported here. Green boxes: |z| > 1.5; red boxes: |z| < −1.5; white boxes: −1.5 < |z| < 1.5.

Biofunctions	Female				Male			
	1 Month TSI		6 Month TSI		1 Month TSI		6 Month TSI	
	7 Gy	7.5 Gy	7 Gy	7.5 Gy	7 Gy	7.5 Gy	7 Gy	7.5 Gy
Concentration of lipid	1.26	−0.46	1.26	−0.46	1.72	1.72	−0.49	1.98
Quantity of amino acids	0.30	0.91	0.30	0.91	−0.61	−0.61	0.61	1.83
Production of reactive oxygen species	2.61	0.43	2.61	0.43	3.34	2.17	−2.61	1.16
Insulin Secretion Signaling Pathway	3.58	0.89	3.58	0.89	3.58	2.68	−3.58	0.00
Synthesis of purine nucleotide	3.00	2.00	3.00	2.00	2.00	1.00	−2.00	2.00
Synthesis of nucleotide	1.79	0.89	1.79	0.89	1.78	0.89	−0.89	2.68
Cellular homeostasis	1.09	−1.81	1.09	−1.81	−1.49	0.27	−1.09	0.41

## Data Availability

All data generated or analyzed during this study are included in this published article and its supplementary information files.

## Supplementary Materials

The following supporting information can be downloaded at: https://www.mdpi.com/article/10.3390/ijms26094227/s1.
