# Supplementary material for: Delayed Impact of Ionizing Radiation Depends on Sex: Integrative Metagenomics and Metabolomics Analysis of Rodent Colon Content
Source: Int J Mol Sci. 2025 Apr 29;26(9):4227. doi: 10.3390/ijms26094227 (PMC12071923; doi:10.3390/ijms26094227)
Supplement: Supplementary file 1 [file ijms-26-04227-s001.zip › AFRRI Gender-ms-suppFigures.pptx]

## Slide 1
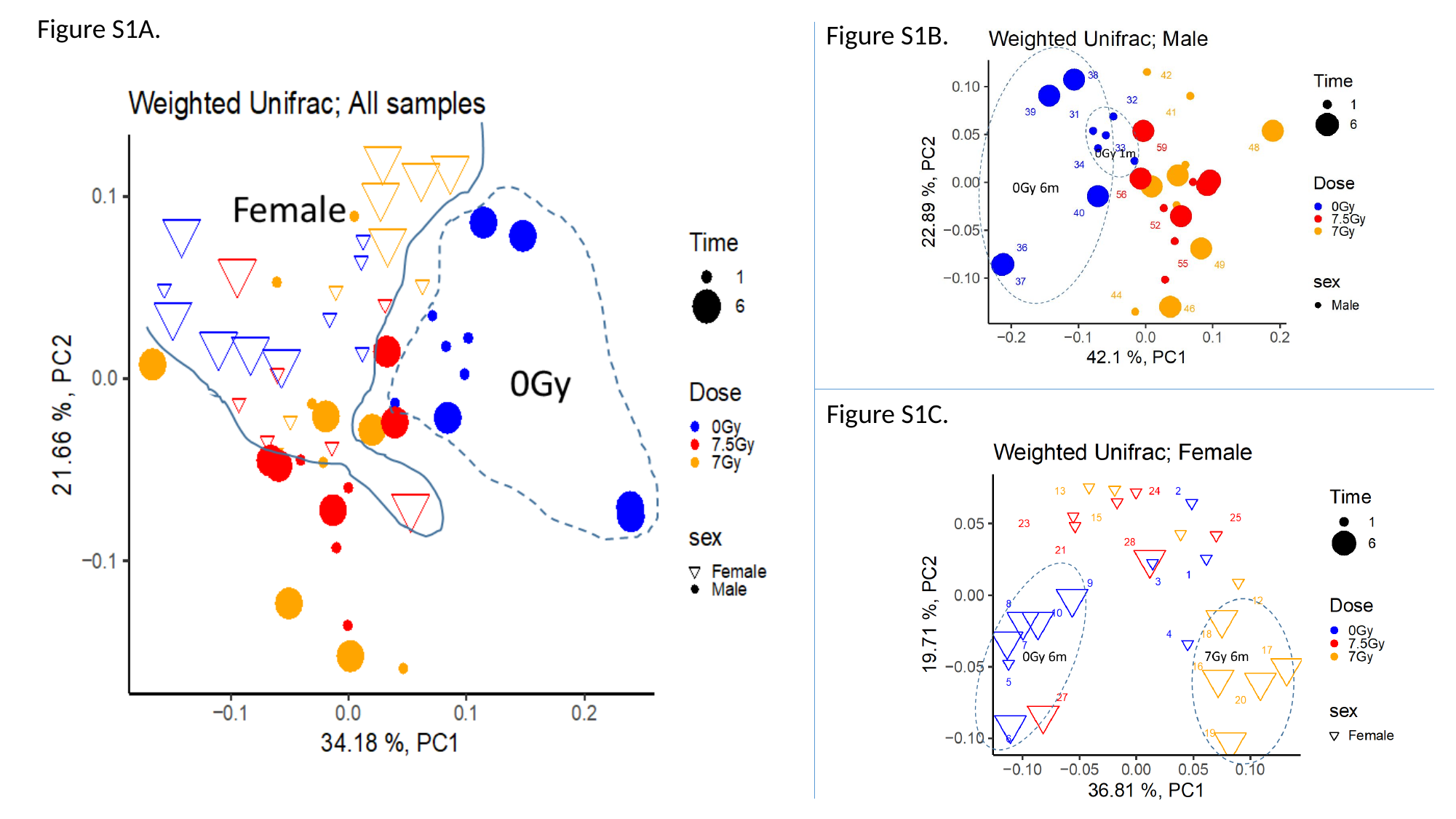

Figure S1A.
Figure S1B.
Figure S1C.

## Slide 2
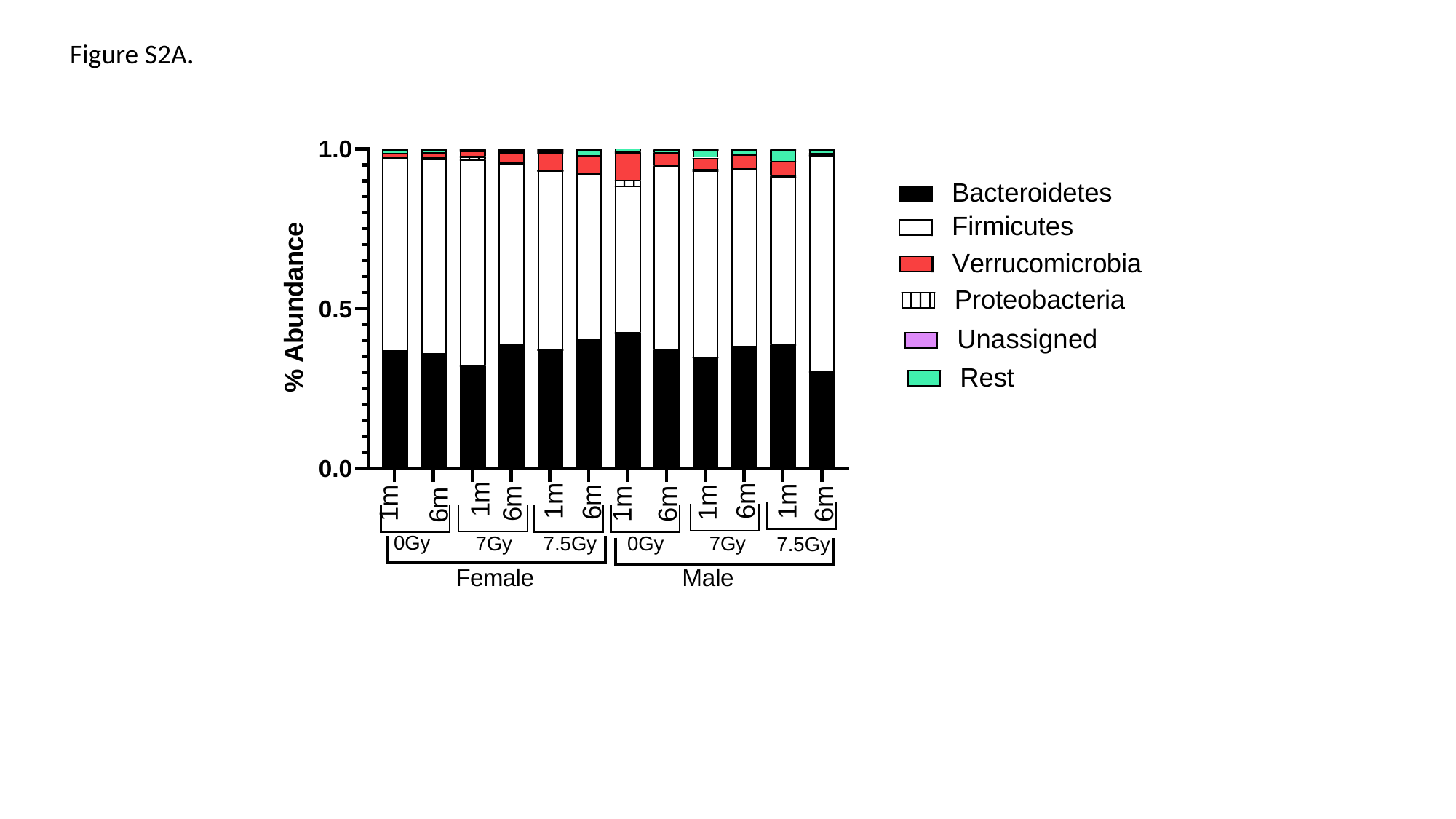

Figure S2A.

## Slide 3
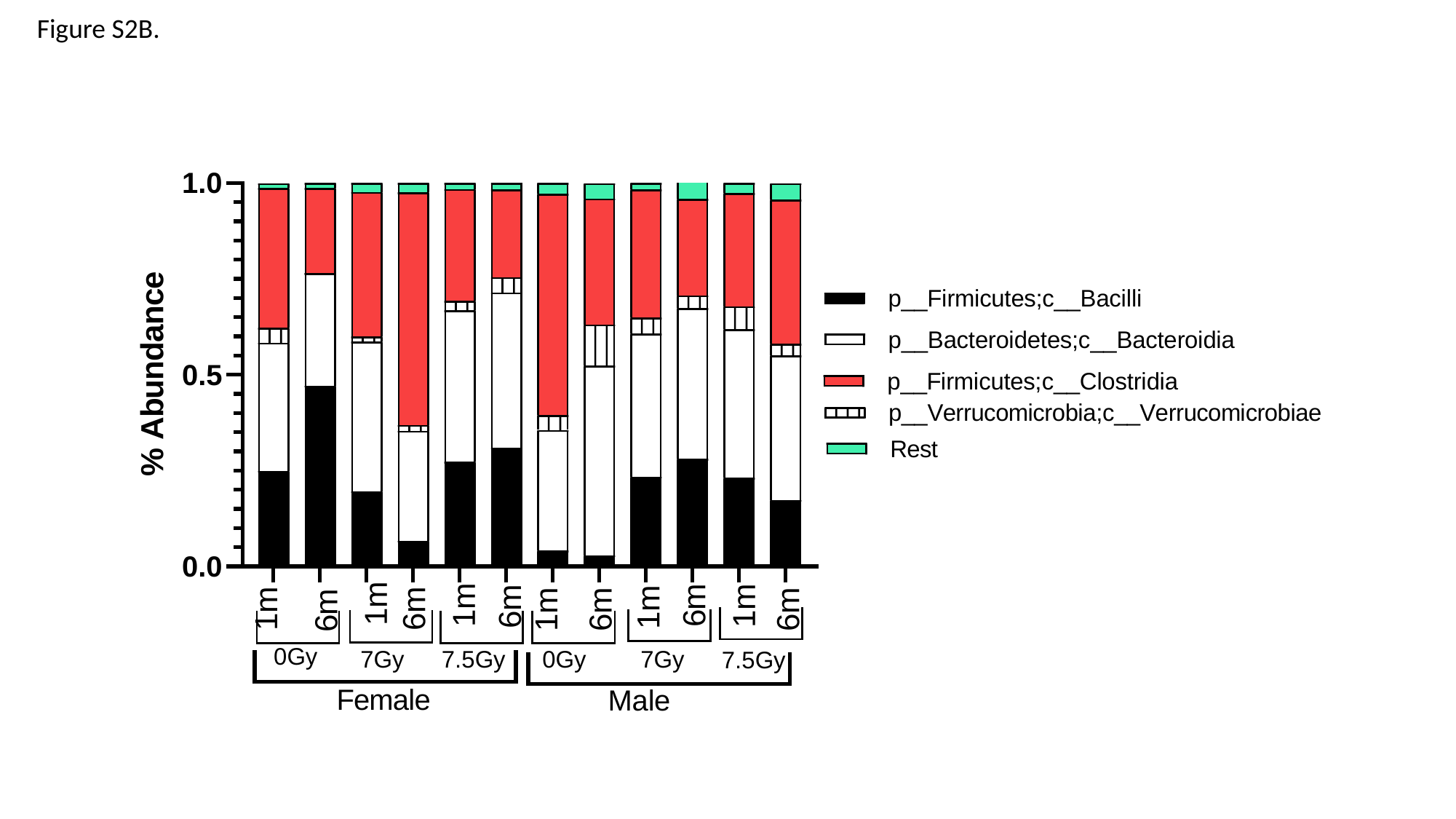

Figure S2B.

## Slide 4
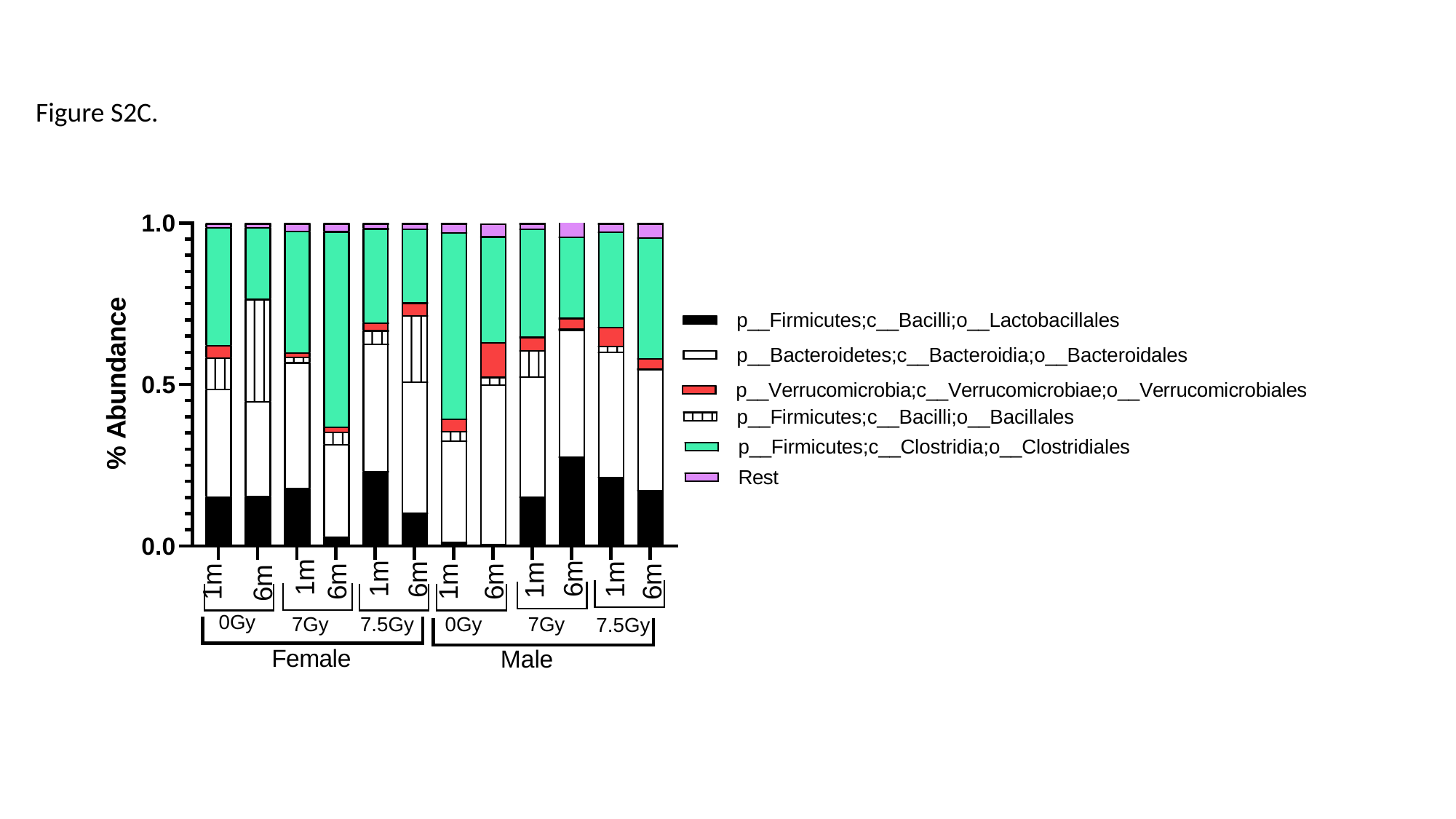

Figure S2C.

## Slide 5
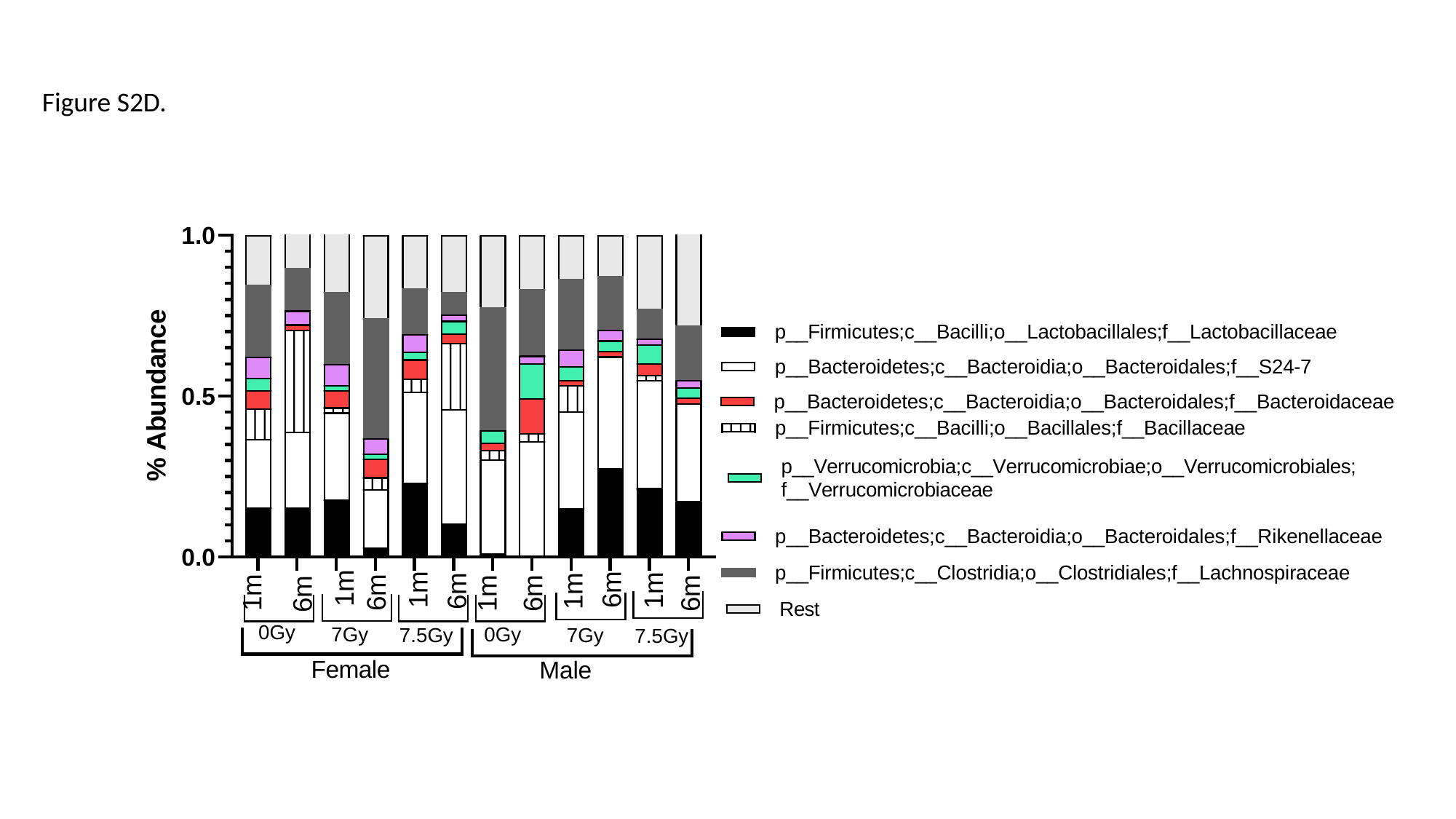

Figure S2D.

## Slide 6
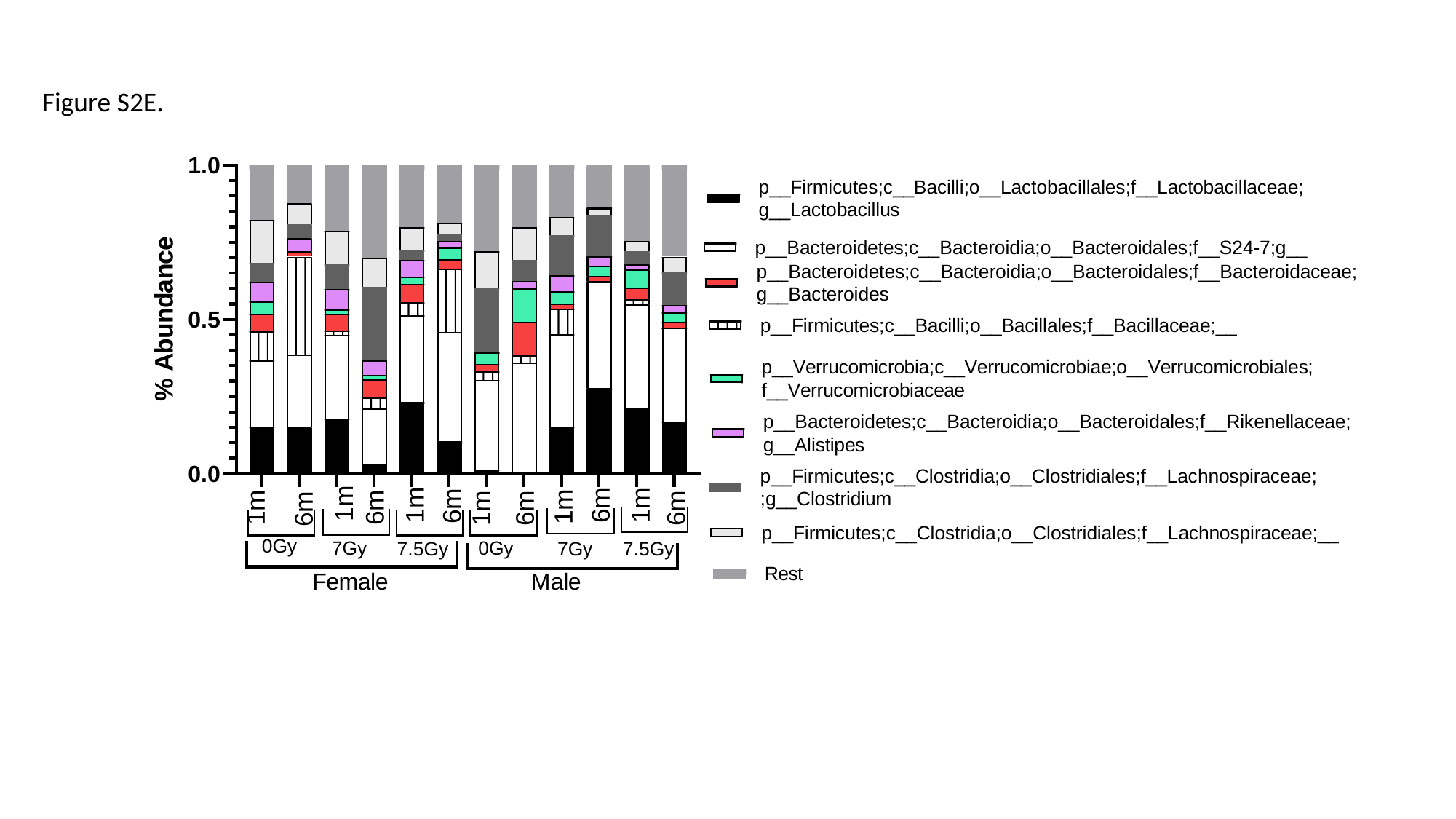

Figure S2E.

## Slide 7
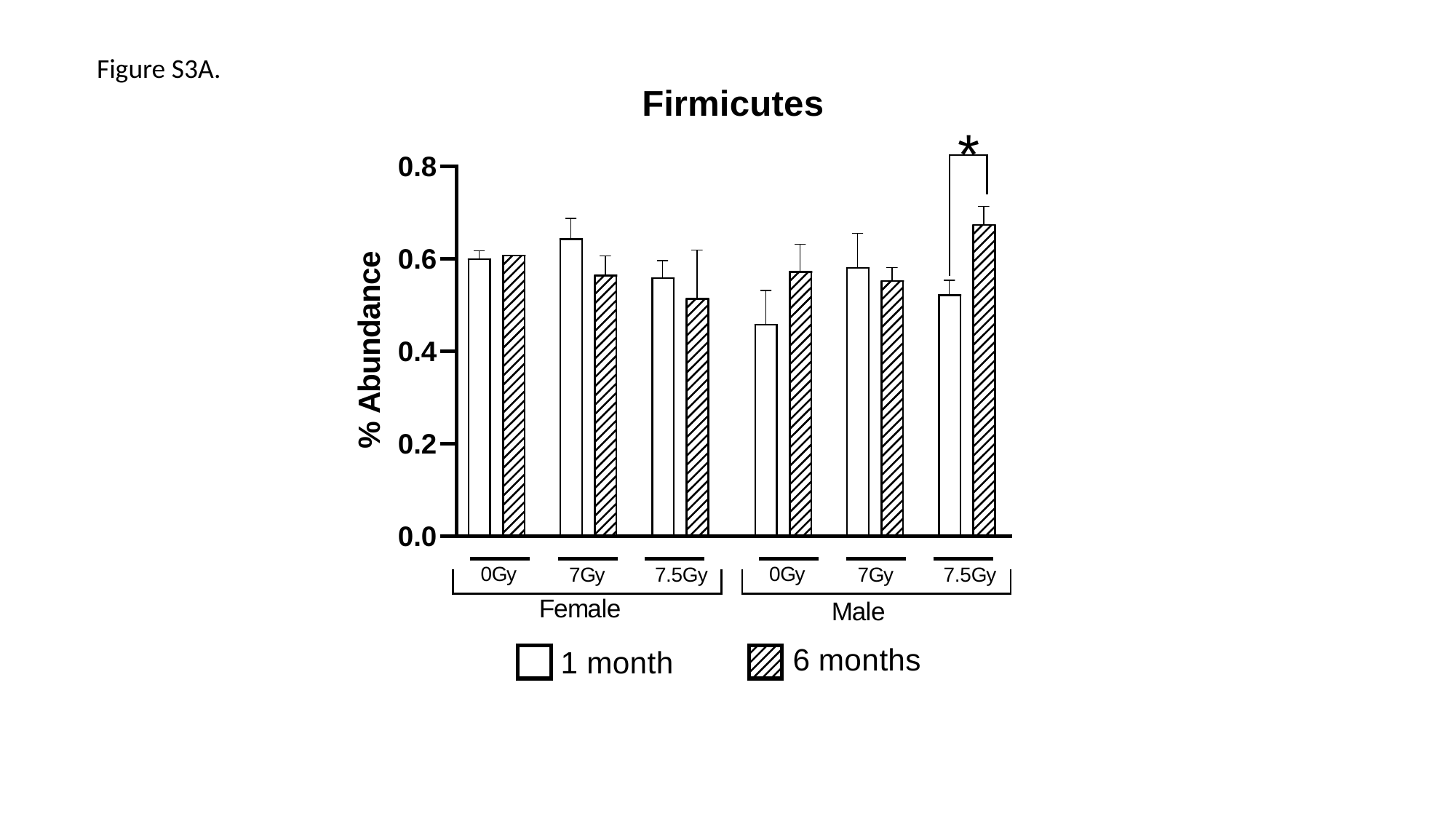

Figure S3A.

## Slide 8
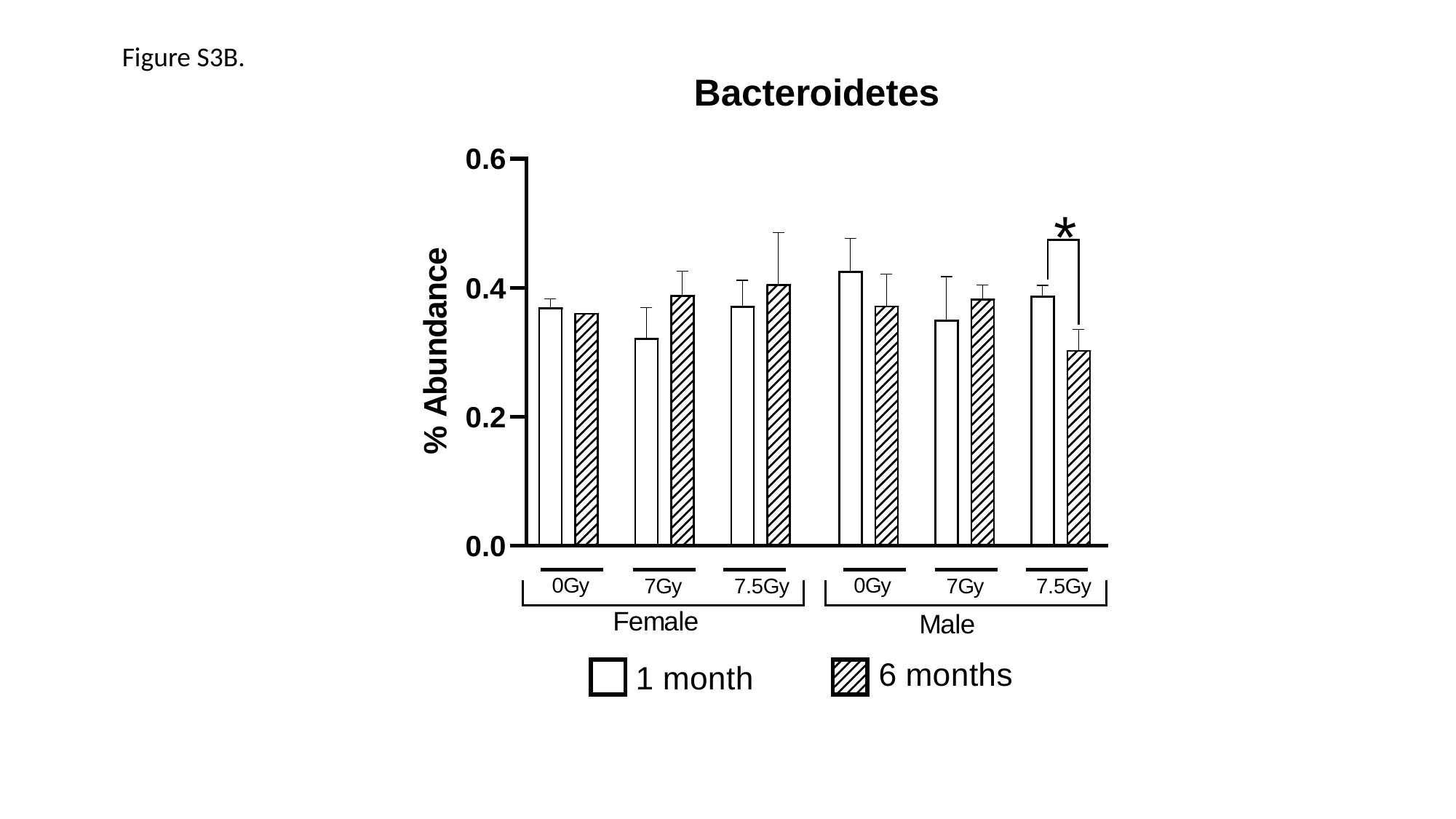

Figure S3B.

## Slide 9
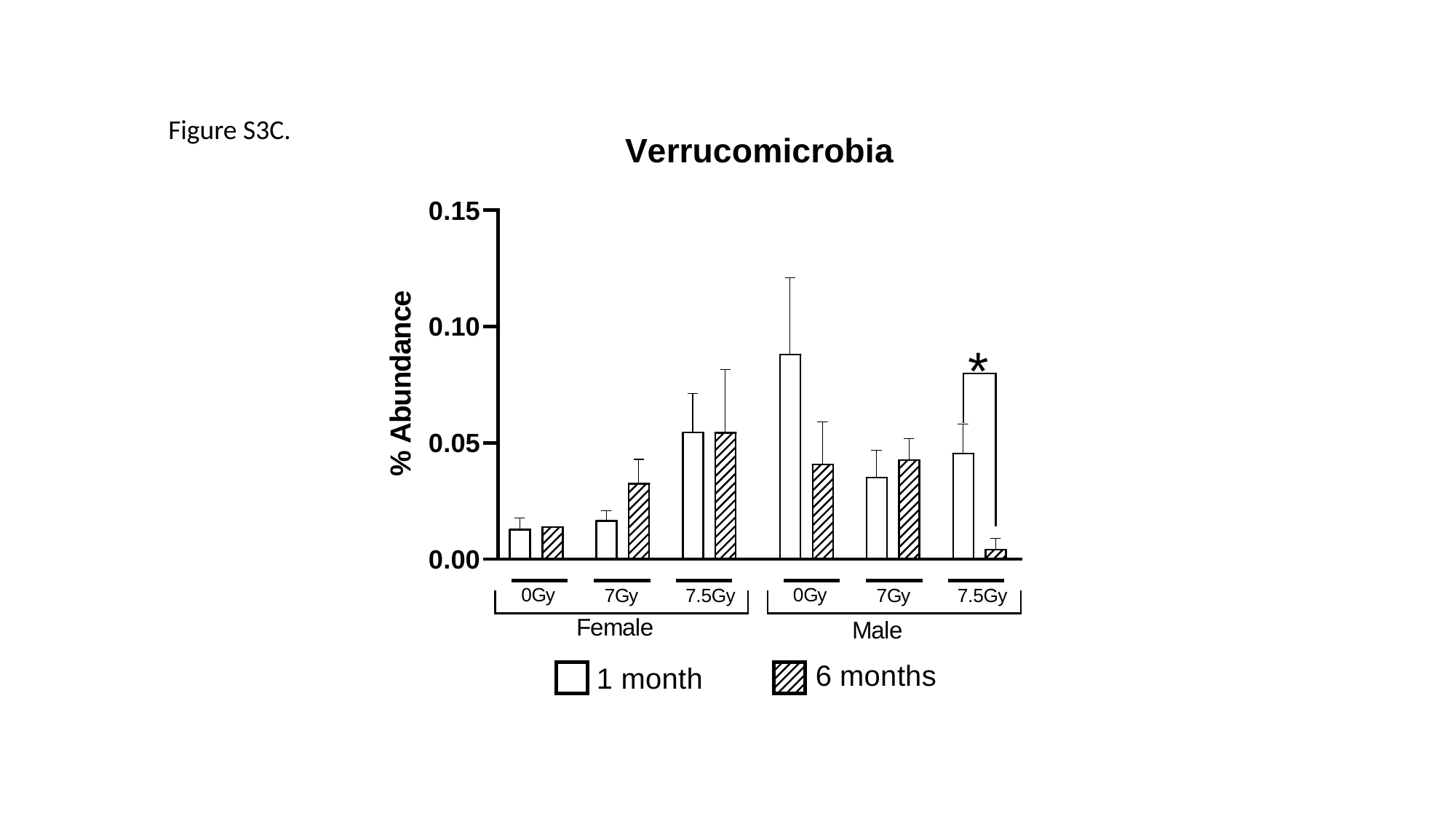

Figure S3C.

## Slide 10
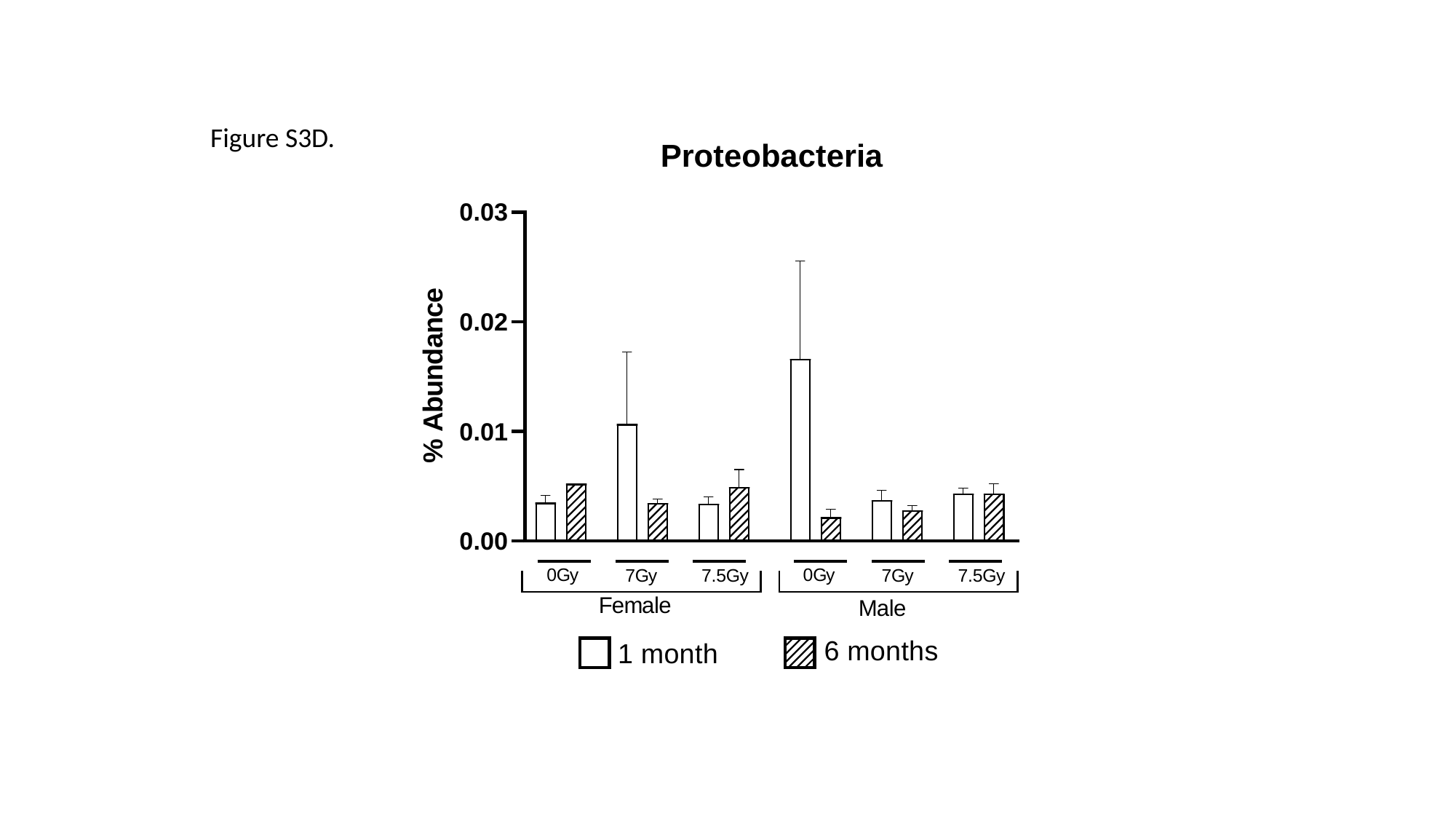

Figure S3D.

## Slide 11
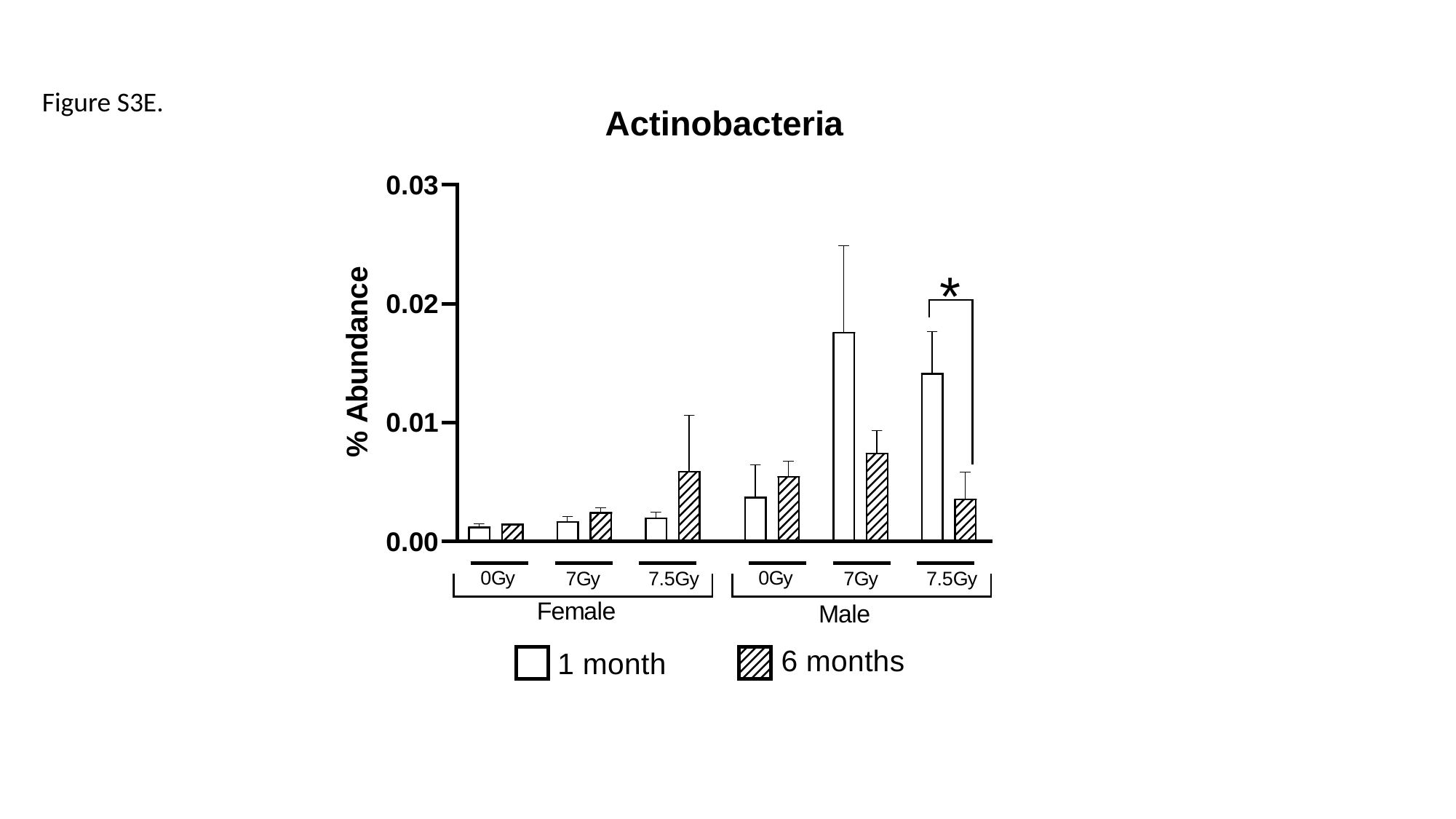

Figure S3E.

## Slide 12
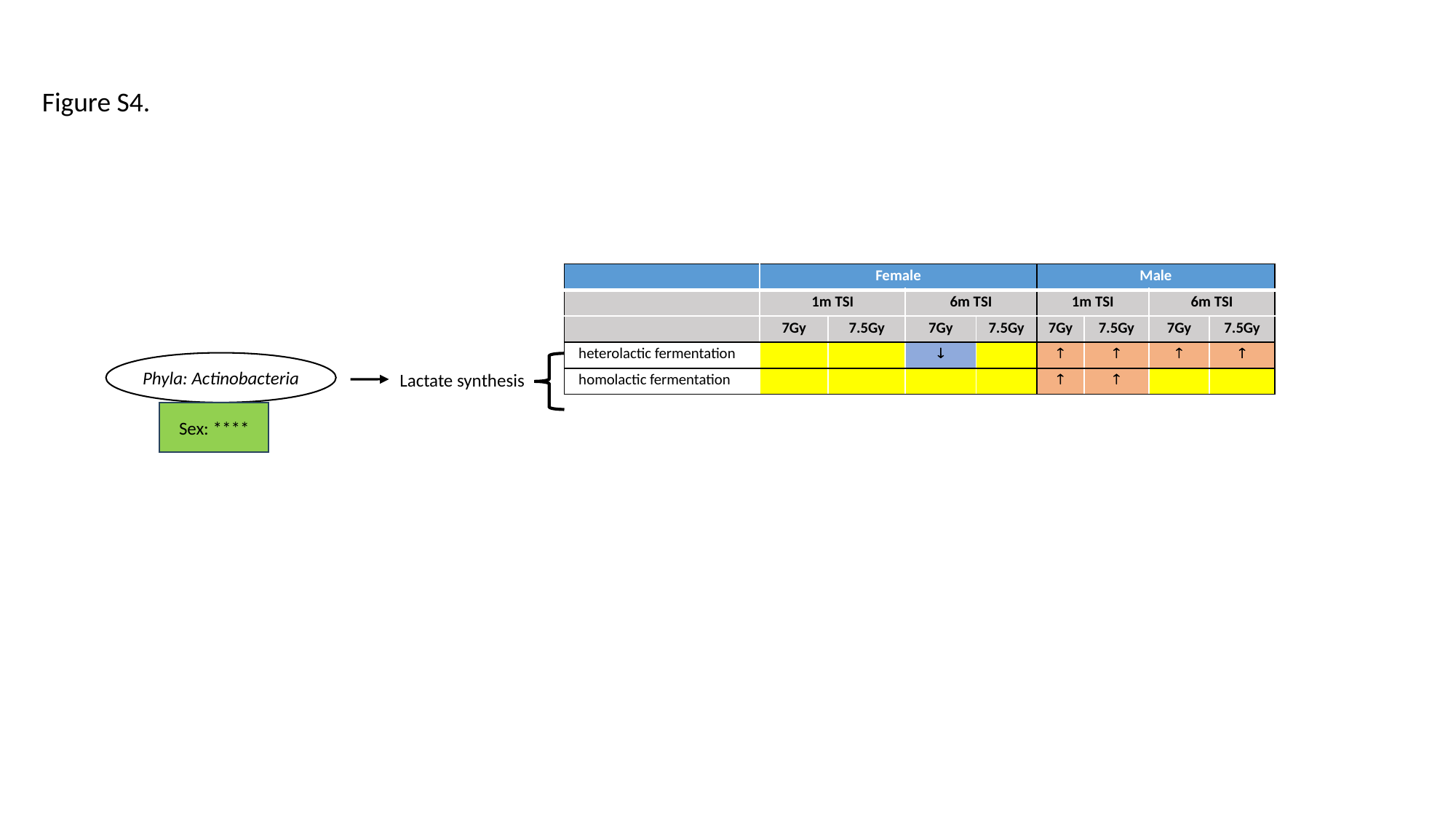

Figure S4.
| | Female | | | | Male | | | |
| --- | --- | --- | --- | --- | --- | --- | --- | --- |
| | 1m TSI | | 6m TSI | | 1m TSI | | 6m TSI | |
| | 7Gy | 7.5Gy | 7Gy | 7.5Gy | 7Gy | 7.5Gy | 7Gy | 7.5Gy |
| heterolactic fermentation | | |  | |  |  |  |  |
| homolactic fermentation | | | | |  |  | | |
Phyla: Actinobacteria
Lactate synthesis
Sex: ****

## Slide 13
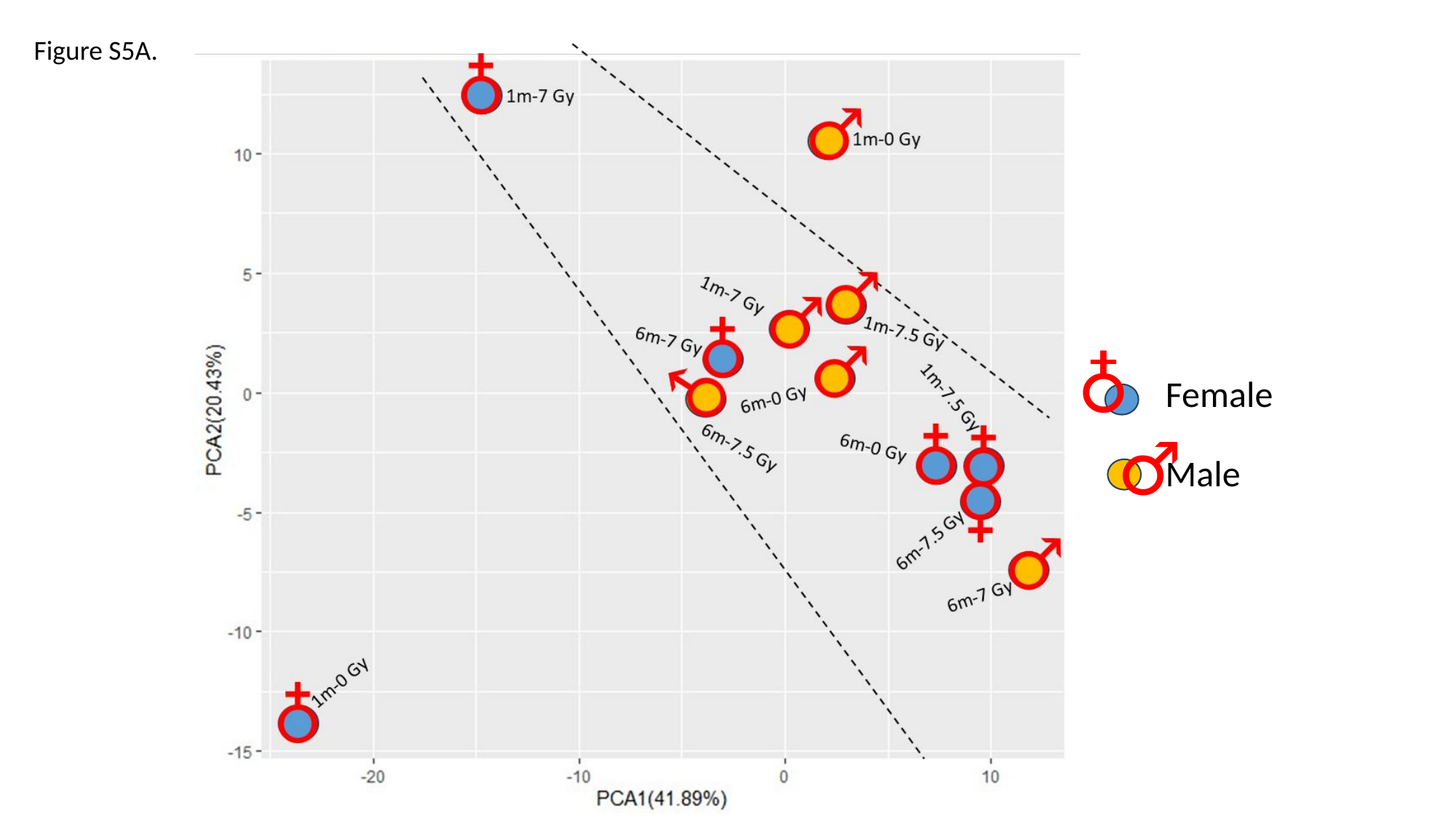

Figure S5A.
♀
Female
Male
♂

## Slide 14
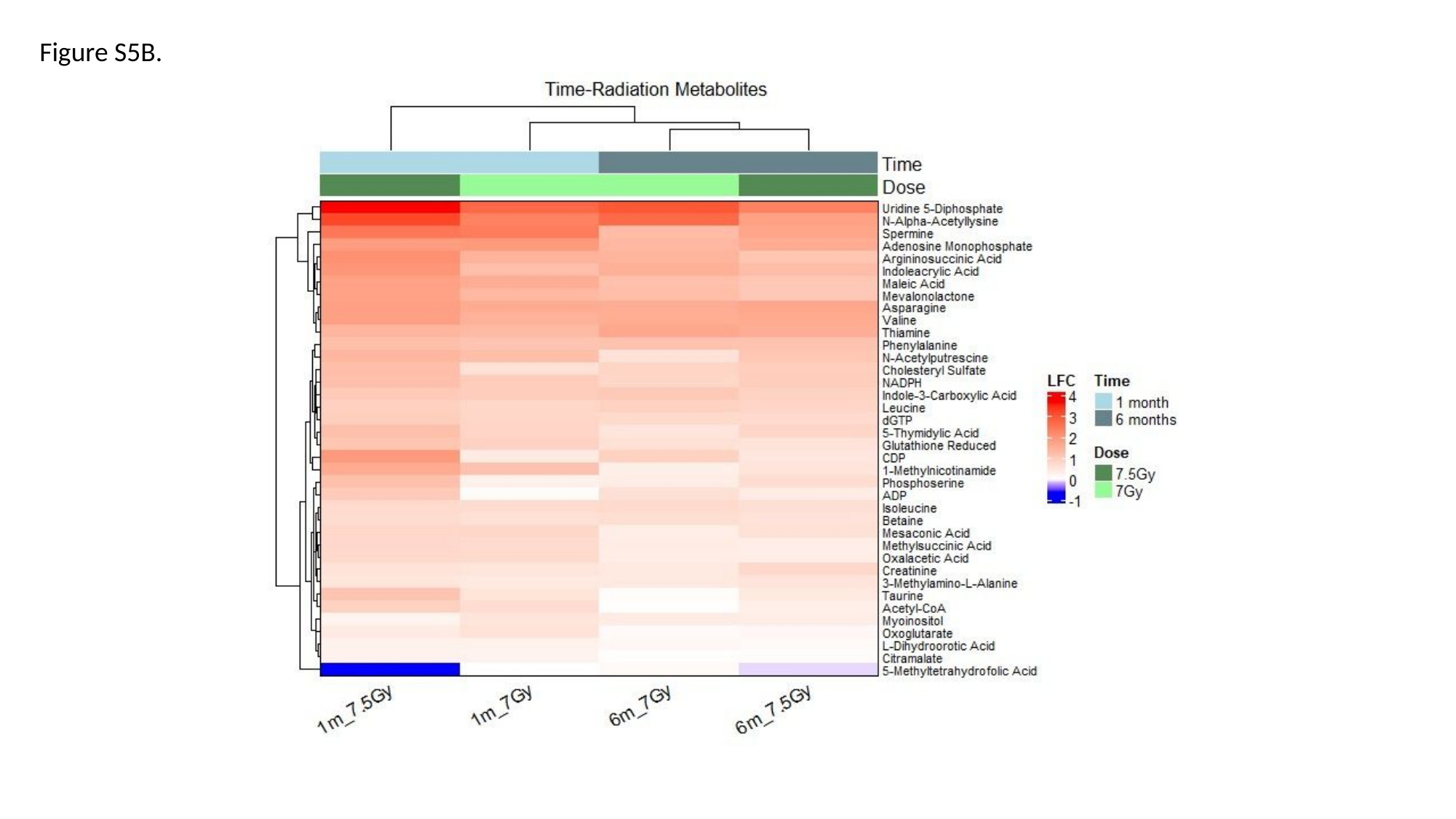

Figure S5B.

## Slide 15
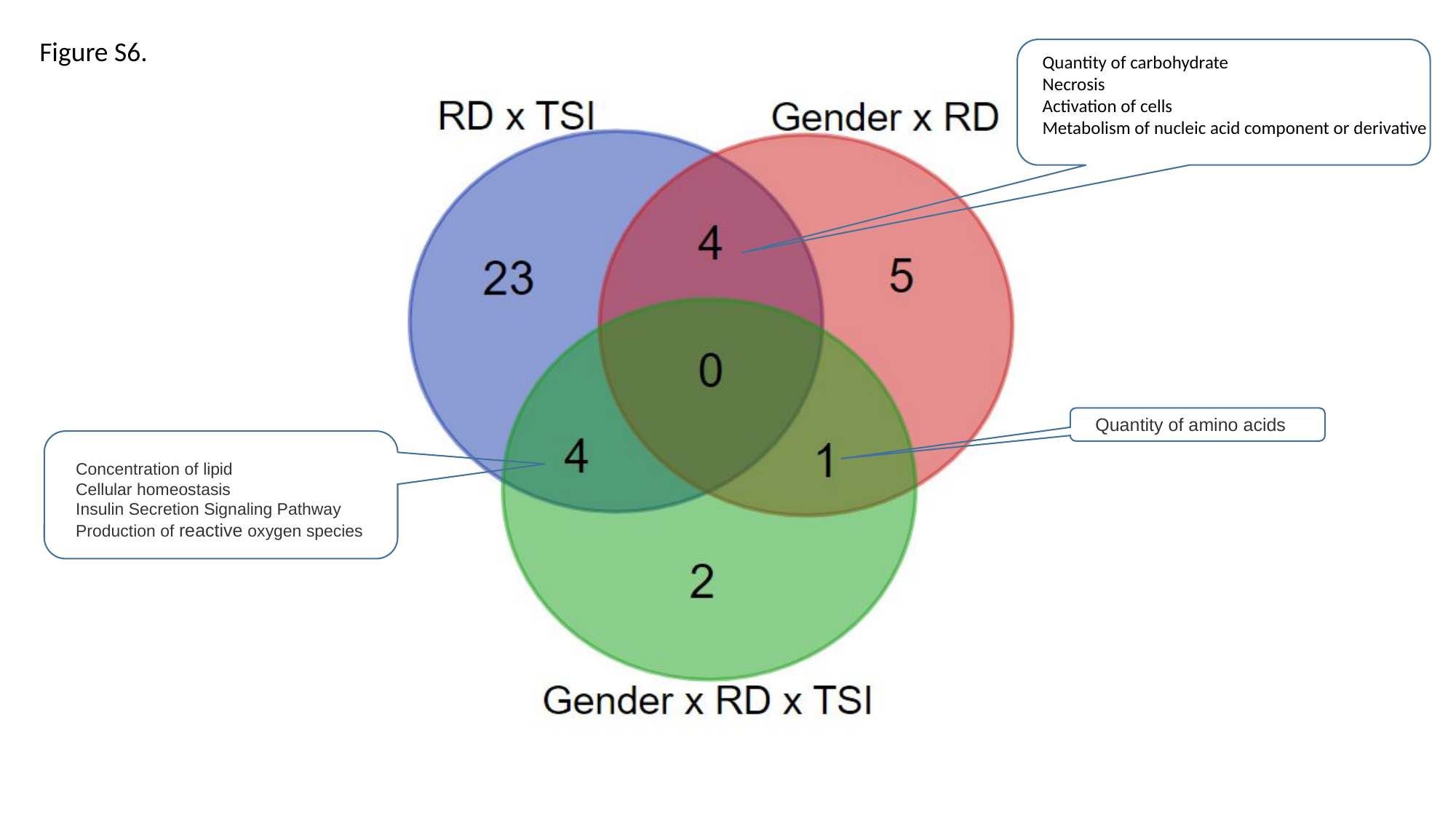

Figure S6.
Quantity of carbohydrate
Necrosis
Activation of cells
Metabolism of nucleic acid component or derivative
Quantity of amino acids
Concentration of lipid
Cellular homeostasis
Insulin Secretion Signaling Pathway
Production of reactive oxygen species
